# Assessing Daily Evapotranspiration Methodologies from One-Time-of-Day sUAS and EC Information in the *GRAPEX* Project

**DOI:** 10.3390/rs13152887

**Published:** 2021-07-23

**Authors:** Ayman Nassar, Alfonso Torres-Rua, William Kustas, Joseph Alfieri, Lawrence Hipps, John Prueger, Héctor Nieto, Maria Mar Alsina, William White, Lynn McKee, Calvin Coopmans, Luis Sanchez, Nick Dokoozlian

**Affiliations:** 1 Department of Civil and Environmental Engineering, Utah State University, Logan, UT 84322, USA;; 2 Utah Water Research Laboratory, Utah State University, Logan, UT 84322, USA; 3 USDA, Agricultural Research Service, Hydrology and Remote Sensing Laboratory, 10300 Baltimore Avenue, Beltsville, MD 20705, USA;; 4 Department of Plants, Soils and Climate, Utah State University, Logan, UT 84322, USA;; 5 USDA, Agricultural Research Service, National Laboratory for Agriculture and Environment, Ames, IA 50011, USA;; 6 Complutum Tecnologías de la Información Geográfica S.L. (COMPLUTIG), 28801 Madrid, Spain;; 7 E. & J. Gallo Winery, Viticulture, Chemistry and Enology, Modesto, CA 95354, USA;; 8 Department of Electrical and Computer Engineering, Utah State University, Logan, UT 84322, USA;

**Keywords:** evapotranspiration (*ET*), daily *ET*, remote sensing, *sUAS*, vineyards, *GRAPEX*, eddy covariance (*EC*), *TSEB*, energy balance

## Abstract

Daily evapotranspiration (*ET*_*d*_) plays a key role in irrigation water management and is particularly important in drought-stricken areas, such as California and high-value crops. Remote sensing allows for the cost-effective estimation of spatial evapotranspiration (*ET*), and the advent of small unmanned aerial systems (*sUAS*) technology has made it possible to estimate instantaneous high-resolution *ET* at the plant, row, and subfield scales. *sUAS* estimates *ET* using “instantaneous” remote sensing measurements with half-hourly/hourly forcing micrometeorological data, yielding hourly fluxes in W/m^2^ that are then translated to a daily scale (mm/day) under two assumptions: (a) relative rates, such as the ratios of *ET*-to-net radiation (*R*_*n*_) or *ET*-to-solar radiation (*R*_*s*_), are assumed to be constant rather than absolute, and (b) nighttime evaporation (*E*) and transpiration (*T*) contributions are negligible. While assumption (a) may be reasonable for unstressed, full cover crops (no exposed soil), the *E* and *T* rates may significantly vary over the course of the day for partially vegetated cover conditions due to diurnal variations of soil and crop temperatures and interactions between soil and vegetation elements in agricultural environments, such as vineyards and orchards. In this study, five existing extrapolation approaches that compute the daily *ET* from the “instantaneous” remotely sensed *sUAS ET* estimates and the eddy covariance (*EC*) flux tower measurements were evaluated under different weather, grapevine variety, and trellis designs. Per assumption (b), the nighttime *ET* contribution was ignored. Each extrapolation technique (evaporative fraction (*EF*), solar radiation (*R*_*s*_), net radiation-to-solar radiation (*R*_*n*_*/R*_*s*_) ratio, Gaussian (*GA*), and Sine) makes use of clear skies and quasi-sinusoidal diurnal variations of hourly *ET* and other meteorological parameters. The *sUAS ET* estimates and *EC ET* measurements were collected over multiple years and times from different vineyard sites in California as part of the USDA Agricultural Research Service Grape Remote Sensing Atmospheric Profile and Evapotranspiration eXperiment (*GRAPEX*). Optical and thermal *sUAS* imagery data at 10 cm and 60 cm, respectively, were collected by the Utah State University *AggieAir sUAS* Program and used in the Two-Source Energy Balance (*TSEB*) model to estimate the instantaneous or hourly *sUAS ET* at overpass time. The hourly *ET* from the *EC* measurements was also used to validate the extrapolation techniques. Overall, the analysis using *EC* measurements indicates that the *R*_*s*_, *EF*, and *GA* approaches presented the best goodness-of-fit statistics for a window of time between 1030 and 1330 PST (Pacific Standard Time), with the *R*_*s*_ approach yielding better agreement with the *EC* measurements. Similar results were found using *TSEB* and *sUAS* data. The 1030–1330 time window also provided the greatest agreement between the actual daily *EC ET* and the extrapolated *TSEB* daily *ET*, with the *R*_*s*_ approach again yielding better agreement with the ground measurements. The expected accuracy of the upscaled *TSEB* daily *ET* estimates across all vineyard sites in California is below 0.5 mm/day, (*EC* extrapolation accuracy was found to be 0.34 mm/day), making the daily scale results from *TSEB* reliable and suitable for day-to-day water management applications.

## 1. Introduction

Evapotranspiration (*ET*) is a key component in the hydro-ecological process, which couples water and energy budgets, links the land surface and the atmosphere [1], and represents water consumption for biomass production [2]. Routine monitoring of actual *ET* is important for a variety of applications, including water resource management, drought monitoring, climate change, and the efficiency of crop irrigation [3–6]. Numerous methods have been used over the past decades to measure *ET*, including lysimeters, Bowen ratio, and eddy covariance (*EC*) flux towers. However, these methods represent limited sampling areas [7], and the measurements are best interpreted for homogeneous surfaces [8]. Spatial techniques are needed to accurately quantify *ET* for improved irrigation scheduling and water management decision support, particularly in complex canopies such as vineyards, which have non-uniform and complex vertical canopy structure, wide and variable row spacing, and deep and complex rooting systems [9]. This canopy structure produces large diurnal changes in solar radiation exposure to soil and plants [9] and requires sophisticated radiation extinction modeling [10,11]. Meanwhile, row spacing ranges between 2.4 m and 3.6 m for vineyards [12], and between 3.6 m and 6 m for orchards trees [13]. Water-limiting conditions across different vineyards in drought-stricken areas, such as California, necessitate the assessment of irrigation demand to set up agricultural water management strategies and decisions [14]. According to the USDA, California produces over 90% of US wine, with a steady growth reaching 635,000 acres [15] in 2019. The high evaporative demand with limited rainfall in the vineyard growing season (May–September), along with the need to achieve grapevine stress targets, constitutes a significant challenge for irrigation scheduling to ensure vineyard productivity [16].

Advances in methods for measuring and modeling the interactions of vineyards with the environment require a better understanding of the processes influencing energy, water, and carbon exchange for highly organized and complex structure perennial crops. Various remote sensing platforms, including satellites, manned aircraft, and small unmanned aerial systems (*sUAS*), improve the potential availability of surface information for estimating *ET* at different spatial scales [17]. However, spatial information from satellites has limitations for *ET* estimation, including spatial and temporal resolutions, the presence of clouds at overpass time, and imagery delivery time [18]. These issues make satellite data challenging to use for the continuous mapping of daily *ET* (*ET*_*d*_) and for real-time irrigation scheduling [19]. However, data fusion methodologies using multiple satellite platforms have improved capabilities for generating daily *ET* on a more routine basis [20,21] and for irrigation scheduling [22]. While manned aircraft have the ability to gather high-resolution data on demand at different times of the day, they are usually cost-prohibitive and, therefore, unlikely to be used to conduct multiple flights over an area of interest [23]. The advent of advanced *sUAS* remote sensing technology with lightweight sensors could overcome some of the previously mentioned remote sensing platform limitations. Compared to satellites, *sUAS* can be described as “flexible in timing”, in that they can be operated as needed at almost any time [7]. Additionally, *sUAS* can provide high spatial and temporal resolution data at sub-meter and multispectral resolutions, although data quality and data processing workflows must be enhanced before *sUAS* can become an efficient data collection platform [24]. Moreover, the areal coverage from *sUAS* is limited compared to satellites. For example, the Landsat 8 scene size is 185 km × 180 km, while an *sUAS* is nearly 1.6 km × 1.6 km, depending on the sensor type and flight height.

Whether using satellite or aerial imagery, the ability to reliably extrapolate from one-time-of-day instantaneous *ET* (*ET*_*i*_) to daily *ET* (*ET*_*d*_) is most useful [25] and relevant for the water management of agricultural crops [3]. Although numerous daily *ET* datasets are available for different applications, these products are often calculated based on the Penman-Monteith approach, the Priestley-Taylor method, or the integration of multiple *ET* estimates at a coarse resolution (≥0.25°) [26]. *EEFLUX* (Earth Engine Evapotranspiration Flux) is another source for obtaining daily *ET* information at 30-m spatial resolution using Landsat data and an energy balance model. However, its temporal resolution of 16 days [27] limits its capability for continuously monitoring *ET* and identifying the spatial variability in irrigation practices that can occur in less than one week. Many current research efforts are being directed towards daily *ET* estimation using surface energy balance models, among them the Two-Source Energy Balance (*TSEB*) model. However, the *TSEB* model provides hourly surface energy fluxes, which requires a scaling/extrapolation approach for generating daily *ET* information. Several studies have compared different daily *ET* methods with an assumption that the ratio of latent heat flux (*LE*) to one energy balance term is constant throughout the day, yet no universal approach has been identified as suitable for all types of land surfaces. Previous studies have indicated that the accuracy of that approach (upscaling daily *ET*) is a function of land surface type. For example, the evaporative fraction (*EF*) approach produced the best agreement in bare soil [28] and soybean [19], while the incoming solar radiation (*R*_*s*_) approach was deemed to be more efficient in estimating daily *ET* in grassland and woody savanna [29]. Another crucial issue for precise daily *ET* estimation is the proper selection of the time-of-day window. In the study conducted by Colaizzi et al. [28], the best time window for extrapolating the hourly *ET* to a daily scale was shown to be within 1 or 2 h of solar noon. This conclusion was also supported by Jackson et al. [30], who identified the time-of-day window for acquiring the *ET* for daily *ET* estimation as within 2 h of solar noon. Therefore, some concerns, such as actual and potential satellite overpass times and cloudiness vs. time of day, should be identified clearly to avoid any error propagation in the daily *ET* estimation.

The need for accurate daily *ET* (*ET*_*d*_) estimates raises two fundamental questions: (1) which daily *ET* extrapolation approach at grapevine row scales can provide reliable values under a variety of crop and environmental conditions and thermal-based *ET* models like *TSEB*? and (2) what time window for acquiring a remotely-sensed *ET* provides the most reliable daily *ET* using an extrapolation approach? Multiple efforts have been made to estimate *ET*_*d*_ for different crops; however, computing *ET*_*d*_ for complex canopies, such as vineyards and grapevine row scales, has not been adequately addressed. In this study, different extrapolation approaches from the literature were assessed for estimating daily *ET* from instantaneous *sUAS ET* estimates for several vineyard sites across California. Specifically, this paper (a) assessed the performance of several daily *ET* extrapolation approaches using *EC* observations and *sUAS* information, and (b) determined an optimal time window for *ET* upscaling from a single to a daily estimate.

### 1.1. Daily ET Upscaling Approaches

*ET* upscaling is commonly performed by assuming conservation of some *ET* metric over the daytime, generally known as a ratio between instantaneous *ET* and a reference variable at a specific time of day, and that nighttime *E* and *T* contributions (soil evaporation and plant transpiration) are negligible or represent some small percentage of the daytime *ET* (on the order of 10%). This hypothesis is commonly known as energy self-preservation [29,31,32] and includes *EF*, *R*_*s*_, and *R*_*n*_*/R*_*s*_ ratio approaches. The second assumption in flux upscaling procedures is that cloud-free conditions persist throughout the daytime [28,33]. However, the clear-sky condition cannot be assured necessarily throughout the season. Other *ET*_*d*_ extrapolation approaches are characterized by a quasi-sinusoidal shape, such as Gaussian (*GA*) and Sine. These approaches assume that the diurnal variation of *ET* is similar to the solar irradiance, with the peak value at solar noon. A description of each approach is presented below.

#### 1.1.1. Evaporative Fraction (*EF*) Approach

One of the most common schemes to extrapolate instantaneous evapotranspiration to a daily value is the evaporative fraction (*EF*) [34]. *EF* is defined as the ratio of latent heat flux (*LE*) to the available energy (the difference between net radiation, *R*_*n*_, and soil heat flux, *G*), assumed to be constant throughout daytime hours. The *EF* approach is presented in Equation (1), as follows:

(1) $$ET_{d}=\left(\frac{LE}{R_{n}-G}\right)\left(\frac{c}{\rho_{w}\lambda }\right){\left(R_{n}-G\right)}_{d}$$

where *ET*_*d*_ is the daily *ET* (mm/day), *LE* is the instantaneous latent heat flux (W/m^2^), *R*_*n*_ is the instantaneous net radiation (W/m^2^), *G* is the instantaneous soil heat flux (W/m^2^), *ρ*_*w*_ is the water density (kg/m^3^), *λ* is the latent heat of vaporization for water (MJ/kg), (*R*_*n*_ – *G*)_*d*_ is the total daily available energy (MJ/m^2^/day), and *c* is a factor equal to 1000 to convert meters to millimeters.

Numerous studies have considered the tendency of the *EF* to be nearly constant during the daytime [35]; however, the combination of soil moisture, weather conditions, topography, and biophysical conditions has an impact on the conservation (or variability) of the *EF* in the daytime [31]. According to Hoedjes et al. [36], self-preservation of the *EF* approach is applicable under dry conditions, while under wet conditions, the *EF* is no longer valid. Nonetheless, a previous study by Crago [32], which used Bowen ratio stations over natural grassland, indicated that, for clear days, the midday *EF* is a good indicator of the daytime average value of the *EF* compared with cloudy days, but the values are still underestimated from the daytime average *EF* due to the concave-up shape of the diurnal variation of the *EF*. This finding is also supported by Li et al. [37], who found that the *EF* is relatively close to the daily average *EF* in the 1000 to 1500 timeframe, and could be used to guide vineyard irrigation practices in arid regions. However, the study by Zhang and Lemeur [38], which used 12 surface network stations called Système Automatique de Mesure de l’Evaporation Rèelle (SAMER) over an area composed of forest (40%) and mixed agriculture (60%), indicated that the *EF* varies during the daytime and could not be used as a guide for *ET*_*d*_ estimates due to factors such as available energy, soil moisture, and other environmental variables. According to the study by Gentine et al. [39], which examined the influence of environmental factors (incoming solar radiation, wind speed, air temperature, soil water content, and leaf area index) on the diurnal behavior of the *EF* over wheat and olive, indicated that *EF* is strongly linked to soil moisture availability and canopy cover. As such, the *EF* increases with increasing the soil moisture and/or fractional cover. On the other hand, they found that the phase difference between net radiation (*R*_*n*_) and the soil heat flux (*G*) must be well-characterized in application models that invoke the *EF* daytime self-preservation.

#### 1.1.2. Solar Radiation (*R*_*s*_) Approach

Another approach for extrapolating *ET*_*i*_ to *ET*_*d*_ is the *R*_*s*_ approach, which is similar to the *EF* but replaces the available energy ((*R*_*n*_ − *G*), instantaneous or daily) term with the incoming solar radiation (*R*_*s*_) as a reference variable. This approach, developed by Jackson et al. [30], assumes that the diurnal *ET* variation is similar to the solar radiation (*ET*~*R*_*s*_), that is, the *ET* is highly correlated and proportional to the *R*_*s*_. Equation (2) demonstrates the expressions for calculating *ET*_*d*_ using the *R*_*s*_ approach.

(2) $$ET_{d}=\left(\frac{LE}{R_{S}}\right)\left(\frac{c}{\rho_{w}\lambda }\right)R_{Sd}$$

where *R*_*sd*_ is the daily solar radiation (MJ/m^2^/day), and *R*_*s*_ is the instantaneous solar radiation (W/m^2^). Other parameters are similar to the *EF* approach.

According to Van Neil et al. [40], the *R*_*s*_ approach is robust when upscaling *ET*_*i*_ to multiple timeframes (e.g., daily, 8-day, and monthly). Moreover, many studies have indicated that solar radiation (*R*_*s*_) is the most robust scalar approach that explains the ratio between the *ET*_*d*_ and *ET*_*i*_ [41].

#### 1.1.3. Ratio of Net Radiation-to-Solar Radiation (*R*_*n*_/*R*_*s*_) Approach

The *R*_*n*_*/R*_*s*_ approach is another approach to scale up *ET*_*i*_ to *ET*_*d*_ using the evaporative fraction (*EF*) and the ratio of net radiation-to-solar radiation (*R*_*n*_*/R*_*s*_) [42]. The *R*_*n*_*/R*_*s*_ approach is presented in Equation (3).


(3) $$ET_{d}=\left(\frac{LE}{R_{n}-G}\right)\left(\frac{R_{n}}{R_{s}}\right)\left(\frac{c}{\rho_{w}\lambda }\right)R_{sd}$$

The parameters of this approach are explained in the *EF* and *R*_*s*_ approaches.

#### 1.1.4. Sine Approach

The Sine approach, developed by Jackson et al. [30], showed that the generic trend of the *ET*_*i*_ during the daylight period is similar to the solar irradiance and could be approximated by a Sine function, where the maximum irradiance occurs at solar noon (~12 p.m.). For cloudy days, the daily *ET* estimates using the Sine approach are less reliable or may be invalid. This implies that the *ET*_*i*_ responds strongly to solar radiation [38]. The approach has been investigated by Zhang and Lemeur [38], who found the Sine approach to be preferable to others for upscaling instantaneous *ET* values.

(4) $$ET_{d}=ET_{i}\left(\frac{2N}{\pi \text{sin}(\pi t/N)}\right)$$

where *ET*_*i*_ represents the instantaneous *ET* (mm/hr), *N* is the total time from sunrise to sunset (*h*) and can be calculated using Equation (5), and *t* is the time elapsed since sunrise (*h*).


(5) $$N=0.945\left\{a+b{\text{sin}}^{2}[\pi (D+10)/365]\right\}$$

In Equation (5), *a* and *b* are latitude-dependent constants, while *D* is the day of the year. For parameters *a* and *b*, Jackson et al. [30] developed a regression model that is a function of the latitude of the location, as shown in Equations (6) and (7), respectively.

(6) $$a=12.0-5.69\times 10^{-2}L-2.02\times 10^{-4}L^{2}+8.25\times 10^{-6}L^{3}-3.15\times 10^{-7}L^{4}$$

and

(7) $$b=0.123L-3.10\times 10^{-4}L^{2}+8.0\times 10^{-7}L^{3}+4.99\times 10^{-7}L^{4}$$

where *L* is the latitude in decimal degrees.

#### 1.1.5. Gaussian (*GA*) Approach

The Gaussian (*GA*) approach has been used recently by Liu et al. [43] to retrieve the *ET*_*d*_ from remotely sensed instantaneous *ET*. The study used *ET*_*i*_ observations from an *EC* system and found that the *ET* diurnal variation follows a Gaussian-fitting curve. When comparing this approach to the Sine and *EF* approaches, results from the study of Liu et al. [43] indicated that *GA* is more accurate using the eddy covariance (*EC*) system.

(8) $$ET_{d}=w\sqrt{\frac{\pi }{2}}\times ET_{i}\times e^{2\left({\left(t_{i}-t_{c}\right)}^{2}/w^{2}\right)}$$

where *w* is the width that equals 2δ, δ is the standard deviation of *ET*_*i*_ values, *t*_*i*_ is the time of the instantaneous *ET* (*ET*_*i*_), and *t*_*c*_ is the time when *ET*_*i*_ arrives at maximum value in the diurnal variation.

### 1.2. Two-Source Energy Balance (TSEB) Model

**Figure 1.**
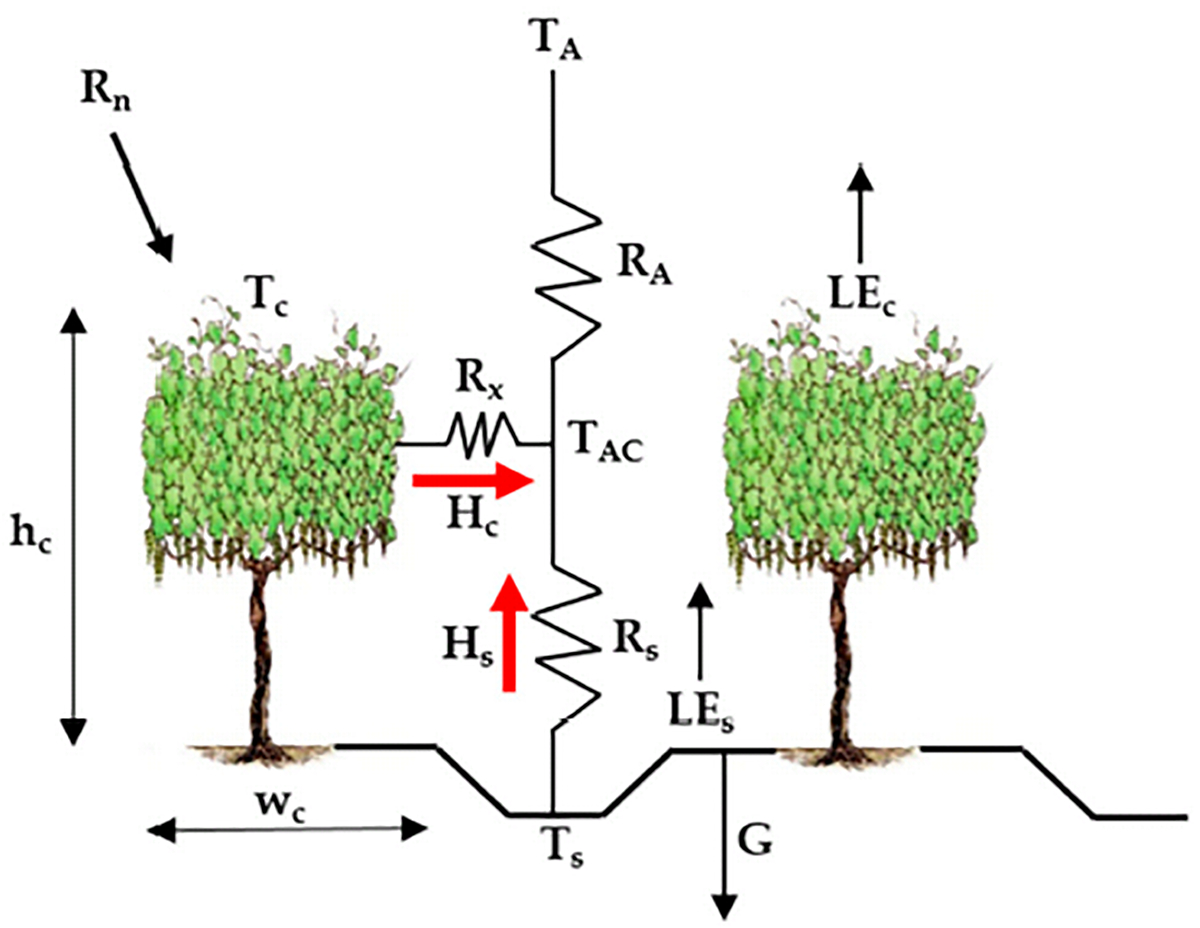
Schematic representation of the Two-Source Energy Balance (*TSEB*) model.

The *TSEB* model was developed by Norman et al. [44] to explicitly accommodate the difference between radiometric and aerodynamic surface temperatures that affect the energy exchange between soil and canopy systems and the lower atmosphere at instantaneous time scales. In the *TSEB* model, turbulent energy fluxes are partitioned between canopy and soil, with different versions applied to separate between those components. These versions include the *TSEB-PT* (Priestly-Taylor), the *TSEB-DTD* (Dual Time Difference), *TSEB-2T-DMS* (Data-Mining Sharpening of temperature), and *TSEB-2T* (Dual Temperature). The *TSEB-PT* version assumes a composite radiometric temperature (*T*_*rad*_) that contains temperature contributions from the soil/substrate and canopy and is decomposed based on the vegetation fractional cover (*f*_*c*_). The *TSEB-DTD* version, developed by Norman et al. [45], uses two observations of *T*_*rad*_: the first observation obtained 1.5 h after the sunrise (*T*_*rad*,*0*_), and the second one during the daytime (*T*_*rad*,*1*_). The *TSEB-DTD* version uses the same approach as *TSEB-PT* to divide the composite *T*_*rad*_ between the soil/substrate and canopy temperatures. Using *TSEB-DTD* could reduce the error in flux estimations when uncertainty exists in local air temperature observations and absolute *T*_*rad*_ [46]. *TSEB-2T-DMS* uses a data-mining fusion algorithm to sharpen the land surface temperature (*LST*), which allows better discrimination between the soil/substrate and canopy temperatures [47]. The *TSEB-2T* approach was originally developed by Kustas and Norman [48] and was further refined and tested by Nieto et al. [49]. The main concept underpinning the *TSEB-2T* approach is to estimate the *T*_*s*_ and *T*_*c*_ from composite *LST* imagery using the relationship between the vegetation index (*VI*) and the *LST* to extract the *T*_*s*_ and *T*_*c*_ within a spatial domain. An early attempt at estimating vineyard water use at a field scale using aerial imagery with *TSEB* and a simple thermal-based contextual scheme suggests the *TSEB* is a robust approach for vineyard *ET* estimation [50]. In this study, the *TSEB* model was used to calculate the instantaneous *ET* at the time of the *sUAS* overpass, and the various schemes were used to extrapolate this one-time-of-day *ET* to a daily value. The *TSEB-2T* model was used for the Sierra Loma vineyard analysis, while the *TSEB-PT* was used for Ripperdan and Barrelli due to limitations in applying the *TSEB-2T* model to those two sites. The average value of the *LAI* was used for these sites, but the *TSEB-2T* requires the *LAI* spatial information to identify the threshold values of *NDVI* of soil, which is based on the empirical relationship between the *NDVI* and *LAI*. More details about the *TSEB-2T* can be found in Nieto et al. [49]. Applying the energy conservation and balance principles, the energy budget in the *TSEB* model can be described in the following equations:

(9) $$R_{n}=LE+H+G,$$

(10) $$R_{nc}=H_{c}+LE_{c},$$

(11) $$R_{ns}=H_{S}+LE_{S}+G,$$

where *R*_*n*_ is the net radiation, and *G* is the soil heat flux. *H* and *LE* are heat fluxes, where *H* is the sensible heat flux and *LE* is the latent heat flux. All flux units are expressed in W/m^2^. Subscripts of *c* and *s* represent the canopy and soil components, respectively. To estimate the sensible heat flux for soil and canopy, Norman et al. [44] proposed a series of soil vegetation resistive schemes (following an analogy with Ohm’s law), as illustrated in Figure 1.

(12) $$H=H_{C}+H_{S}=\rho_{air}C_{p}\frac{T_{AC}-T_{A}}{R_{A}}=\rho_{air}C_{p}\left[\frac{T_{C}-T_{AC}}{R_{x}}+\frac{T_{S}-T_{AC}}{R_{S}}\right]$$

where *ρ*_*air*_ is the air density (kg/m^3^), *C*_*p*_ is the heat capacity of the air at constant pressure (J/kg/K), *T*_*A*_ is the air temperature (Kelvins), *T*_*c*_ and *T*_*s*_ are the canopy and soil temperatures (Kelvins), respectively, and *T*_*AC*_ is the temperature of the canopy air space (Kelvins), which is calculated with Equation (13).

(13) $$T_{AC}=\frac{\frac{T_{A}}{R_{a}}+\frac{T_{C}}{R_{x}}+\frac{T_{S}}{R_{S}}}{\frac{1}{R_{A}}+\frac{1}{R_{x}}+\frac{1}{R_{S}}}$$

where *R*_*A*_ is the aerodynamic resistance to heat transport from the soil/canopy system, *R*_*x*_ is the boundary layer resistance of the canopy leaves, and *R*_*s*_ *is* the aerodynamic resistance to heat transport in the boundary layer close to the soil surface. All resistances are expressed in (s/m). The mathematical expressions used to compute the resistance network are detailed in Equations (14)–(16).

(14) $$R_{A}=\frac{\text{ln}\left(\frac{z_{T}-d_{0}}{z_{0M}}\right)-\Psi_{h}\left(\frac{z_{T}-d_{0}}{L}\right)+\Psi_{h}\left(\frac{z_{0M}}{L}\right)}{\kappa^{\prime }u_{*}}$$

(15) $$R_{x}=\frac{C^{\prime }}{LAI}\sqrt{\left(\frac{l_{w}}{U_{d_{0}+z_{0M}}}\right)}$$

(16) $$R_{S}=\frac{1}{c{\left(T_{S}-T_{A}\right)}^{1/3}+bu_{S}}$$

where u_*_ is the friction velocity, calculated as the following:

(17) $$u_{*}=\frac{\kappa^{\prime }u}{\text{ln}\left(\frac{z_{u}-d_{0}}{z_{0M}}\right)-\Psi_{m}\left(\frac{z_{u}-d_{0}}{L}\right)+\Psi_{m}\left(\frac{z_{0M}}{L}\right)}$$

In Equation (17), *z*_*u*_ and *z*_*T*_ are the measurement heights for wind speed (*u*) and air temperature (*T*_*A*_), respectively, *d*_0_ is the zero-plane displacement height, and *z*_0*M*_ is the roughness length for momentum. The unit of *z*_0*M*_ is expressed in m. In the *TSEB* model versions, the roughness length of momentum (*z*_0*M*_) is assumed to equal the roughness length for heat transport (*z*_0*H*_), as the aerodynamic resistance of the canopy elements (*R*_*x*_) already takes into account the different efficiencies between momentum and heat transport. *κ*′ represents the von Karman’s constant, which is equal to 0.4. Ψ_*h*_ and Ψ_*m*_ are the adiabatic correction factors for heat and momentum, respectively. The details of these two factors are described in Brutsaert [51]. In Equation (15), *C*′ is assumed to be 90 s^1/2^/m and *l*_*w*_ represents the average width of leaf (m). The coefficients (*b* and *c*) in Equation (16) depend on the turbulent length scale in the canopy, the soil-surface roughness, and the turbulence intensity in the canopy. More details can be found in the work by Nieto et al. (2019a), Nieto et al. (2019b), Kustas et al., and Kondo and Ishida [11,49,52,53].

## 2. Methodology

### 2.1. Study Area

**Figure 2.**
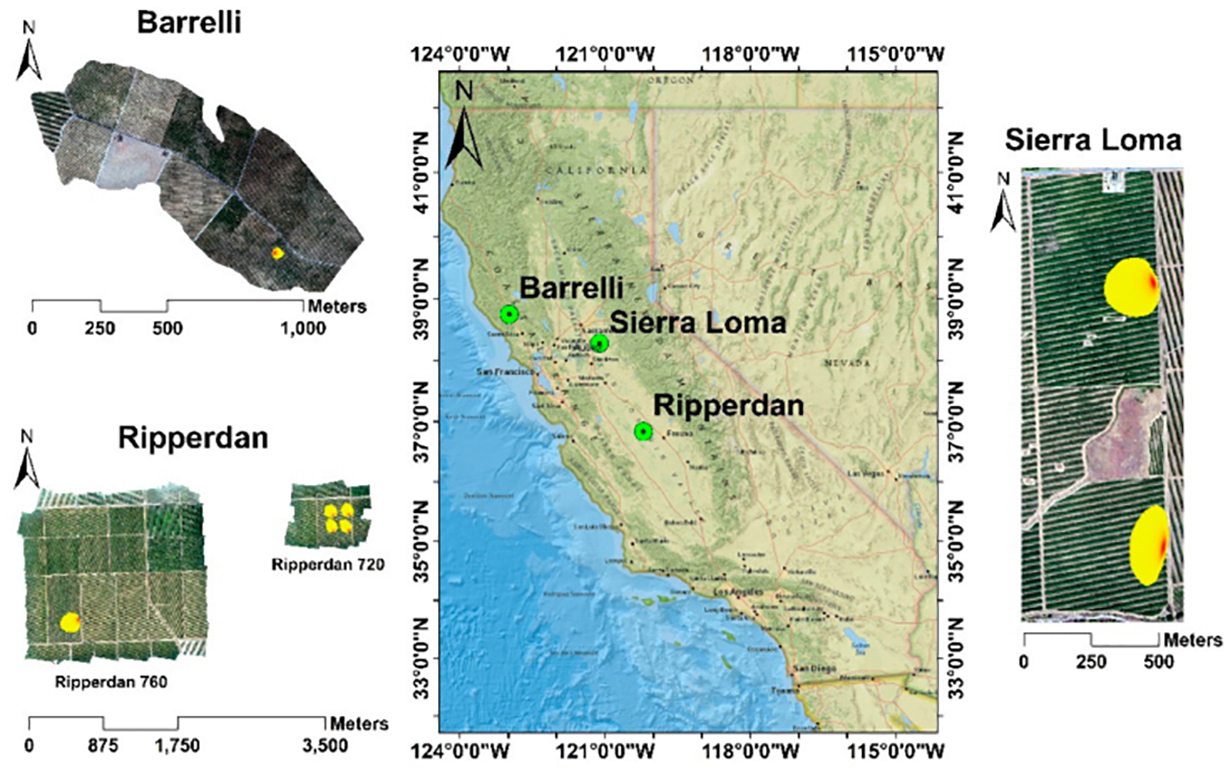
Layout of study vineyards in Central Valley, California with estimated typical flux footprint/source area for the *EC* towers.

The experiment was conducted within three different climate regions located in California, as shown in Figure 2. All of these sites are part of the Grape Remote Sensing Atmospheric Profile and Evapotranspiration eXperiment (*GRAPEX*) project [54], led by the USDA ARS in collaboration with E&J Gallo Winery, University of California in Davis, Utah State University, NASA, and others. The overall objective of the *GRAPEX* project is to provide the vineyard manager and grower with spatially distributed, remotely sensed *ET* information for improving irrigation water use efficiency and detecting crop stress in multiple vineyard blocks. This would facilitate water conservation efforts in California’s Central Valley, which has been experiencing frequent and severe drought conditions. The project began in 2013 at two pinot noir blocks located within the Sierra Loma Vineyard near Lodi, California (38.29°N, 121.12°W) in Sacramento County (see Figure 2) [7]. The two vineyard blocks, north and south, differed in maturity and age, having been implemented in 2009 and 2011, respectively. The configuration of the trellising system in both fields is the same, with vine trellises 3.35 m apart and an east–west orientation. In 2017, the *GRAPEX* project extended the observations to include two additional vineyards: Barrelli vineyard (38.75°N, 122.98°W), located near Cloverdale, California, and Ripperdan vineyard (36.84°N, 120.21°W), located near Madera, California. With the expansion of the *GRAPEX* project from Sierra Loma to the Barrelli site to the north and Ripperdan to the south, a large range in trellis designs, climate regions, vine varieties, canopy structure, and vine physiology are represented. The Ripperdan vineyard was planted in 2009, whereas the Barrelli vineyard was implemented in 2010. Both the Barrelli and Ripperdan vineyards employ different plantation structures and vine varieties. The vine rows in Barrelli have a northeast–southwest row orientation, with a row spacing of 3.35 m and predominately Cabernet Sauvignon vine variety, while in Ripperdan, the row direction is east–west, with a row spacing of 2.74 m growing Chardonnay and Merlot. Data collection campaigns/intensive observation periods (*IOPs*) in these sites were conducted in the veraison period (from mid-July to early August), when the crop evaporative demand increases.

### 2.2. Procedure

**Figure 3.**
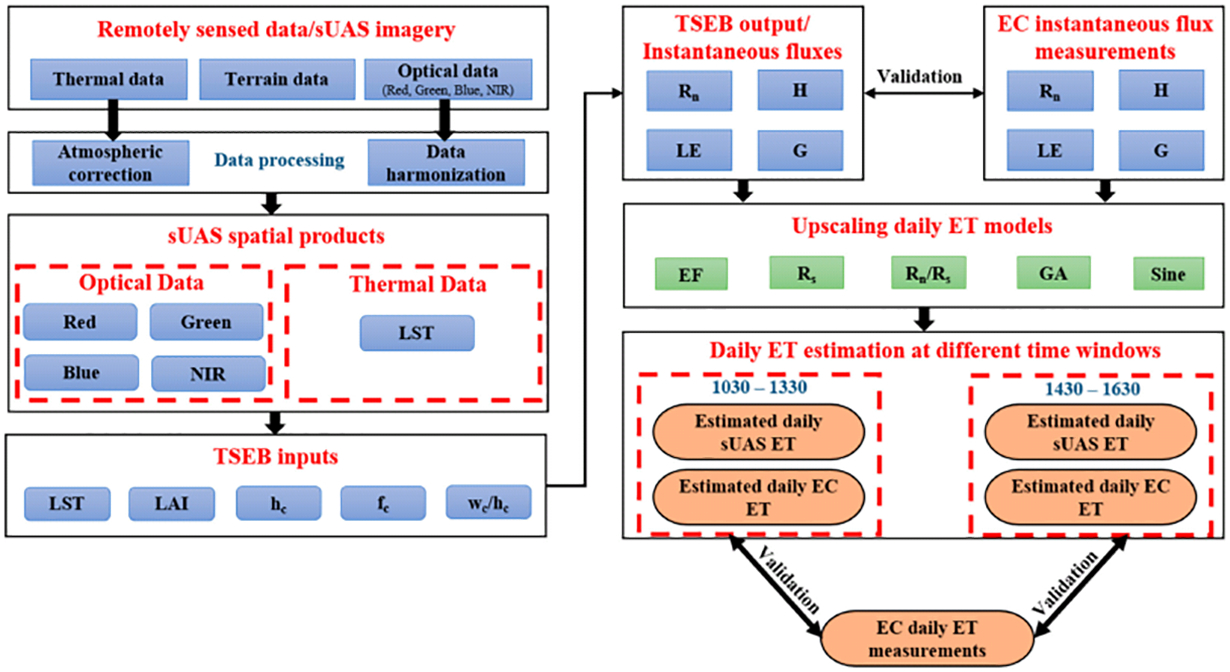
Study methodology for assessing different upscaling daily *ET* methods in *sUAS*.

Figure 3 illustrates the procedure used for this study. First, available eddy covariance (*EC*) flux tower data was filtered to select cloud-free days only. Then, five different *ET*_*d*_ approaches were applied to the *LE* fluxes from the *EC* measurements for upscaling the *ET* to the daily timescale. The analysis was performed using *EC* observations at different vine phenological stages (April–May, June–August, and September–October). Finally, daily *sUAS ET* information, produced using the *TSEB* model, and results from the five approaches for upscaling/extrapolating the daily *ET* were compared against the measured *ET*_*d*_ from the *EC* tower data. Two time windows were selected for the daily *ET* estimation: the first was near solar noon (1030–1330), and the second was in the afternoon (1430–1630). The reasons for these selections were (a) satellite overpass time, (b) *sUAS* flexibility, which allows for flights at different hours, including mid to late afternoon, and (c) an opportunity to assess the suitability of using later (2+ hours after solar noon) *sUAS* flights for the estimation of daily *ET*.

#### 2.2.1. sUAS Data Processing

The *AggieAir sUAS* Program at Utah State University (https://uwrl.usu.edu/aggieair/ accessed on 10 December 2020) [55] acquired high-resolution imagery at 450 m above ground level (*agl*), resulting in visible and near-infrared data at a 0.10 m spatial resolution, and a thermal spatial resolution at 0.6 m. The spectral range of the visible and near-infrared data was similar to Landsat; however, the thermal band range was wider, with a bandwidth spanning from 7 to 14 μm. Thermal data was acquired using a radiometrically calibrated micro-bolometer camera. Table 1 lists the information concerning the different *AggieAir sUAS* flights. In this study, the obtained *sUAS* images were georectified using ground control points (*GCPs*). Details of the optical and thermal information are presented below.

##### Thermal Data

Changes in the transmissivity and atmospheric radiance can adversely affect the *sUAS* thermal data [56]. Details about thermal data calibration can be found in the work by Torres-Rua [56], while the work by Torres-Rua et al. [57] shows that the *TSEB* model is insensitive to surface emissivity. The *AggieAir sUAS* Program has a thermal protocol to use over 90% of overlap for thermal raw imagery collected after *sUAS* launching but before mission data collection upon internal lens temperature stabilization of the microbolometer camera. These two steps address potential vignetting as well as the temperature drifting effect observed in other *sUAS* applications.

##### Optical Data

Radiometric agreement between different remote sensing platforms is important for further integration. An internal evaluation of the optical data obtained from different *sUAS* flights was performed by aggregating the high-resolution imagery up to Landsat scale using a point-spread function (PSF). The resulting 30-m pixels were found to agree with Landsat reflectance information. This is due to the use of different sensors than the ones used by Hassan-Esfahani et al. [58].

#### 2.2.2. Eddy Covariance (*EC*) Fluxes

Surface energy fluxes (*LE* and *H*) were calculated from the *EC* measurements of the sonic temperature, water vapor, and vertical wind speed. In this study, the measurements obtained from the *EC* were averaged over a 60-min time interval to ensure appropriate averaging time for calculating the *H* and *LE*. The sensible heat flux was calculated from the product of the air density, the specific heat of air, and the covariance between the vertical wind speed and sonic temperature. The expression used to calculate *H* is shown in Equation (18).

(18) $$H=\rho_{a}C_{p}\left(\bar{{U_{z}}^{\prime }{T_{s}}^{\prime }}\right)$$

where *ρ*_*a*_ is the air density (kg/m^3^), *C*_*p*_ is the heat capacity of the air at constant pressure (J/kg/K), *U*_*z*_′ is the vertical wind speed (m/s), and *T*_*s*_′ is the sonic temperature (Kelvins).

The latent heat flux (*LE*) was calculated from the product of the latent heat of vaporization (*λ*) and the covariance between the vertical wind speed (*U*_*z*_′) and the water vapor density (*ρ*_*v*_′). The formula used to calculate the *LE* is illustrated in Equation (19).

(19) $$LE=\lambda \left(\bar{{U_{z}}^{\prime }{\rho_{v}}^{\prime }}\right)$$

where *ρ*_*v*_′ is the water vapor density (kg/m^3^).

Table 2 describes the *EC* towers installed at the different vineyard sites to monitor *ET*. The *EC* measurements (April to October) obtained are the surface energy fluxes (*R*_*n*_, *H*, and *LE*) and micrometeorological data. More details about the in situ micrometeorological measurements can be found in the work by Nassar et al. [7].

In Sierra Loma, each *EC* tower monitors grapevines of different ages, while 4 flux towers in Ripperdan 720 measure different water management approaches at 4 different blocks. In this study, the footprint analysis of each *EC* tower was performed to validate the results obtained from the *TSEB* model. The Kljun et al. [59] model was used for describing the fetch of the *EC* contribution area for the hourly period encompassing the *sUAS* flight times. The shape and orientation of the *EC* footprint depend on multiple micro-meteorological conditions that are observed by the *EC* towers installed at the sites, which include the friction velocity, wind speed, wind direction, roughness length, standard deviation of the crosswind velocity, and Monin–Obukhov length as well as the *EC* tower height. In this study, the authors did not include any energy balance closure to the *EC* information to minimize biases.

### 2.3. Goodness-of-Fit Statistics

#### 2.3.1. Quantitative Statistics

The performance indices to evaluate the daily *ET* approaches in this study involved comparisons of the modeled *ET* from the five different approaches against daily *ET* measurements from the *EC* towers. Computed statistical metrics included the root mean square error (*RMSE*), mean absolute error (*MAE*), mean absolute percentage error (*MAPE*), Nash–Sutcliffe efficiency coefficient (*NSE*), and the coefficient of determination (*R*^*2*^). The *NSE* coefficient checks the capability of the model to reproduce the following statistical components: correlation coefficient of (*r*), mean (*μ*), and variance (*s*). *NSE* values range between −∞ and 1, where 1 represents a perfect agreement, while a value of 0 means that the model results are not better than the average of the variable of interest, and values < 0 indicate unacceptable model performance [60].

(20) $$\text{RMSE}=\sqrt{\frac{\sum_{i=1}^{N}{\left(O_{i}-P_{i}\right)}^{2}}{N}}$$

(21) $$\text{MAE}=\frac{\sum_{i=1}^{N}\left|O_{i}-P_{i}\right|}{N}$$

(22) $$\text{MAPE}=\frac{\sum_{i=1}^{N}\left|\frac{O_{i}-P_{i}}{O_{i}}\right|\times 100}{N}$$

(23) $$\text{NSE}=1-\frac{\sum_{i=1}^{N}{\left(O_{i}-P_{i}\right)}^{2}}{\sum_{i=1}^{N}{\left(O_{i}-\bar{O}\right)}^{2}}$$

(24) $$R^{2}={\left[\frac{\sum_{i=1}^{N}\left(O_{i}-\bar{O}\right)\left(P_{i}-\bar{P}\right)}{\sqrt{\sum_{i=1}^{N}{\left(O_{i}-\bar{O}\right)}^{2}}\sqrt{\sum_{i=1}^{N}{\left(P_{i}-\bar{P}\right)}^{2}}}\right]}^{2}$$

where *O*_*i*_ denotes the observed value, *P*_*i*_ denotes the modeled value, $\bar{O}$ denotes the mean observed value, $\bar{P}$ denotes the mean modeled value, and *N* represents the number of observations.

#### 2.3.2. Graphical Representations

Different graphical representations were used to visualize and evaluate the datasets from the *EC* towers and the performance of the extrapolation techniques. Boxplots were created to describe the variance of surface energy fluxes (*R*_*n*_, *H*, *LE*, and *G*) at each hour in the dataset. Boxplots were also used to evaluate the performance of the five daily *ET* extrapolation schemes by presenting the distribution of relative error at each individual hour during the daytime, as shown in the Appendix. Moreover, scatterplots were used to compare the modeled fluxes from *TSEB* and the measurements from *EC* systems to evaluate model performance.

## 3. Results and Discussion

### 3.1. Diurnal Variation of Energy Fluxes from EC Measurements

**Figure 4.**
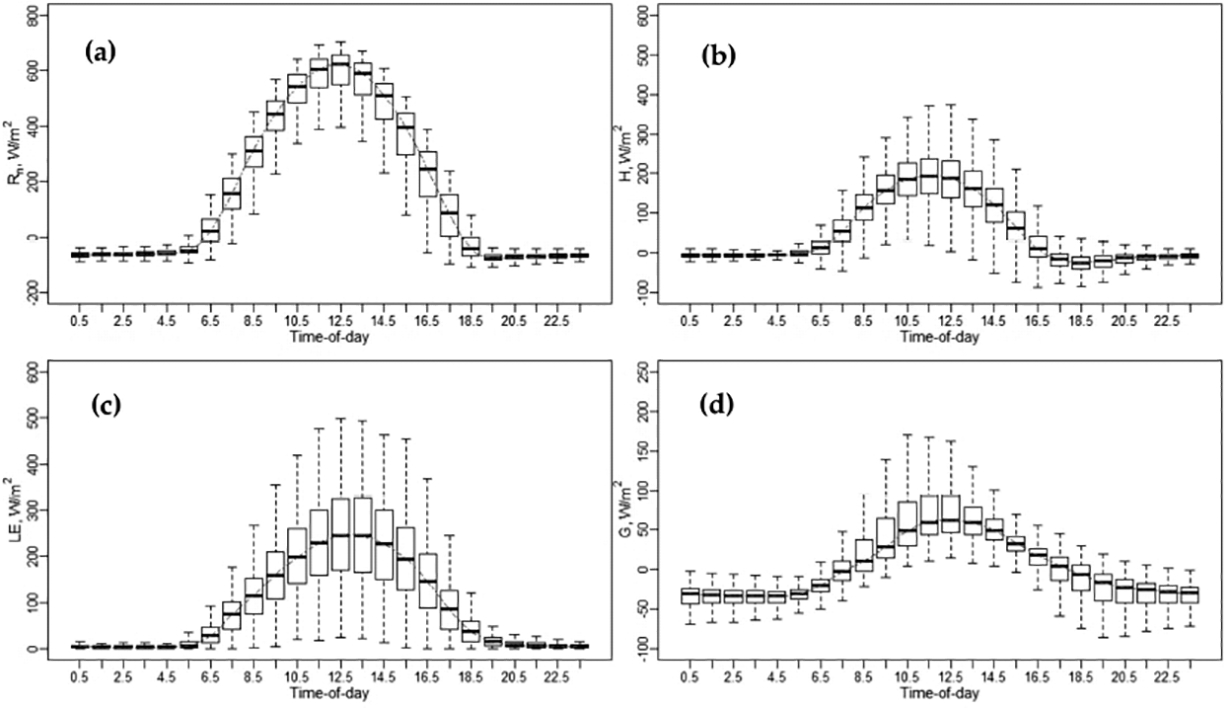
Diurnal variations of energy fluxes at Sierra Loma Sites 1 and 2 for the years 2014 to 2018, from the April to October irrigation season. (**a**) Net radiation (*R*_*n*_), (**b**) sensible heat flux (*H*), (**c**) latent heat flux (*LE*), (**d**) soil heat flux (*G*).

An example of the diurnal variation of surface energy fluxes (*R*_*n*_, *H*, *LE*, and *G*) is shown in Figure 4 for the Sierra Loma vineyard. Diurnal variation plots for the other vineyard study sites (Ripperdan 760, Ripperdan 720, and Barrelli) are shown in Appendices B.1, C.1 and D.1 The boxplot at each individual hour represents the seasonal variation (April to October) of surface fluxes due to changes in the irrigation scheduling and variations in weather conditions (wind speed, air temperature, vapor pressure deficit, and soil moisture) [61]. Overall, the behavior of *R*_*n*_ diurnal variation is similar among the different sites, as the solar radiation is relatively consistent. As shown in Figure 4, *R*_*n*_ values are negative in the nighttime and late evening. In the daytime, *R*_*n*_ values vary, with maximum values of nearly 700 W/m^2^ at solar noon depending on the daily solar radiation. The diurnal pattern of *R*_*n*_ is almost systematic with a peak value appearing during midday, around 1200 standard time. The diurnal distribution of both *H* and *LE* exhibits a typical concave-down shape, with minimums in the early morning and late afternoon. The peak value appears near solar noon, between 1030 and 1330. Overnight, the *H* is almost negative, while the *LE* is approximately equal to zero, as the incoming solar radiation (*R*_*s*_) value is 0 at night. Although this is not always the case, the approximation may be acceptable for night [62]. In summertime, the *LE* value overnight is very small and rarely exceeds 5–10% of the daily total [63]. The study by Shapland et al. [64], which was conducted to estimate the *ET* over vineyards in California, assumed that the turbulent fluxes are zero during the night to avoid the uncertainty associated with the flux measurement. Another study by Tolk et al. [65], which aimed to quantify the nighttime evapotranspiration *ET*_*N*_-to-24-h *ET* (*ET*_*24*_) of irrigated and dryland cotton in a semiarid climate, indicated that the ratio of *ET*_*N*_-to-*ET*_*24*_ ranged from an average of 3% for a dryland cotton crop to around 7% for irrigated alfalfa. The contribution of *ET*_*N*_-to-*ET*_*24*_ was the result of a relatively high nighttime vapor pressure deficit (*VPD*) and wind speed.

Flux observations indicated that the *LE* values were higher than the *H* across the different vineyards, as shown in Figure 4 and Appendices B.1, C.1 and D.1. These results stem from the fact that the vineyards are drip irrigated and, during most of the growing season, the cover crop is senescent, so *ET* is largely controlled by the vine canopy and, hence, mainly affected by the vine leaf stomatal conductance. The diurnal variation of soil heat flux (*G*) does not follow symmetric behavior, having a right skewness. As demonstrated in Figure 4 and Appendices B.1, C.1 and D.1, the *G* value is much lower than other energy fluxes (*R*_*n*_, *H*, and *LE*), where the peak does not persist across different vineyard sites. For overnight and later evening, *G* is negative and could yield values around −100 W/m^2^, as shown in Figure 4d at Sierra Loma vineyard, with similar results obtained at the other vineyard sites included in this study. In the energy balance, usually, the *G* value is estimated as a portion of *R*_*n*_ (~0.35 *R*_*n*_) for remote sensing *ET* models. Meanwhile, the *G* value is highly affected by the *LAI*, canopy architecture, row direction, and trellis design, as well as the incoming solar radiation. Reducing the canopy fractional cover results in an increased daytime soil heat flux (*G*), while increasing the areal coverage of vegetation leads to decreased soil heat flux and greater above-canopy latent heat fluxes, as long as there is ample root zone soil moisture to meet the atmospheric demand.

**Figure 5.**
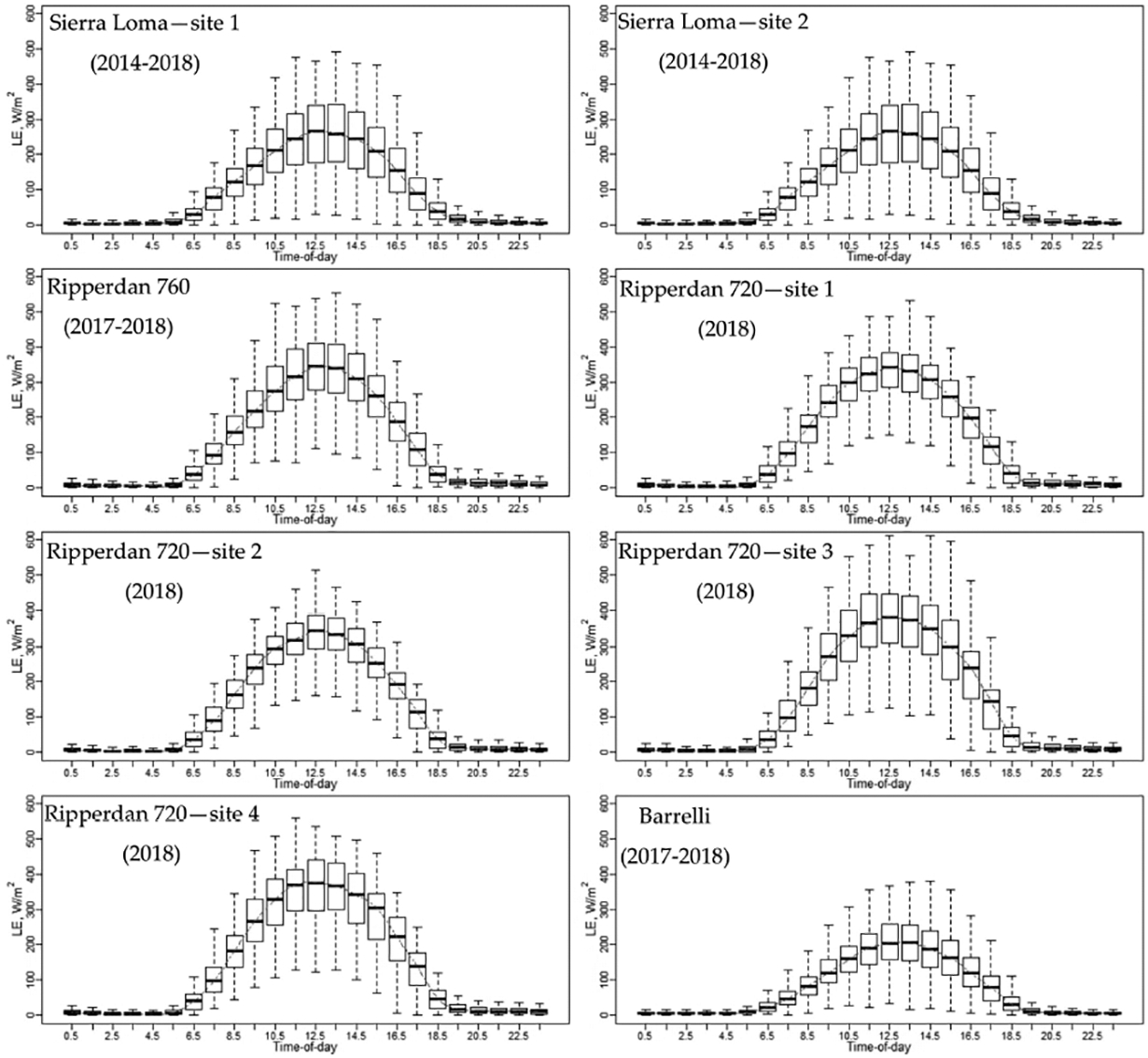
Diurnal variations of *LE* for each *EC* included in this study for the years 2014 to 2018, from the April to October irrigation season.

Figure 5 shows the *LE* diurnal variation at each individual *EC* tower included in this study. The boxplot at every hour represents the seasonal variation from April to October due to weather changes and irrigation scheduling. Overall, the general temporal trend of the *LE* has a shape that resembles solar radiation at different vineyard sites, with a peak value near solar noon, between 1030 and 1330. In early morning and overnight, the *LE* values were close to zero. Comparing the diurnal variation of *LE* at different vineyards, the Barrelli site had the lowest *LE* values. The Barrelli vineyard is located near the Pacific Coast shoreline, which brings cool maritime air that cools the warm interior valleys. The cool and moist air over Barrelli is associated with a decrease in the vapor pressure deficit (*VPD*) and more cloudiness, which causes a decrease in *ET* demand. In Sierra Loma and Ripperdan, the *VPD* and air temperature were higher than Barrelli, as both sites are exposed to a warm Mediterranean climate, which is characterized by abundant sunshine and a large day-to-night temperature difference and, therefore, increases the *ET* demand [66].

**Figure 6.**
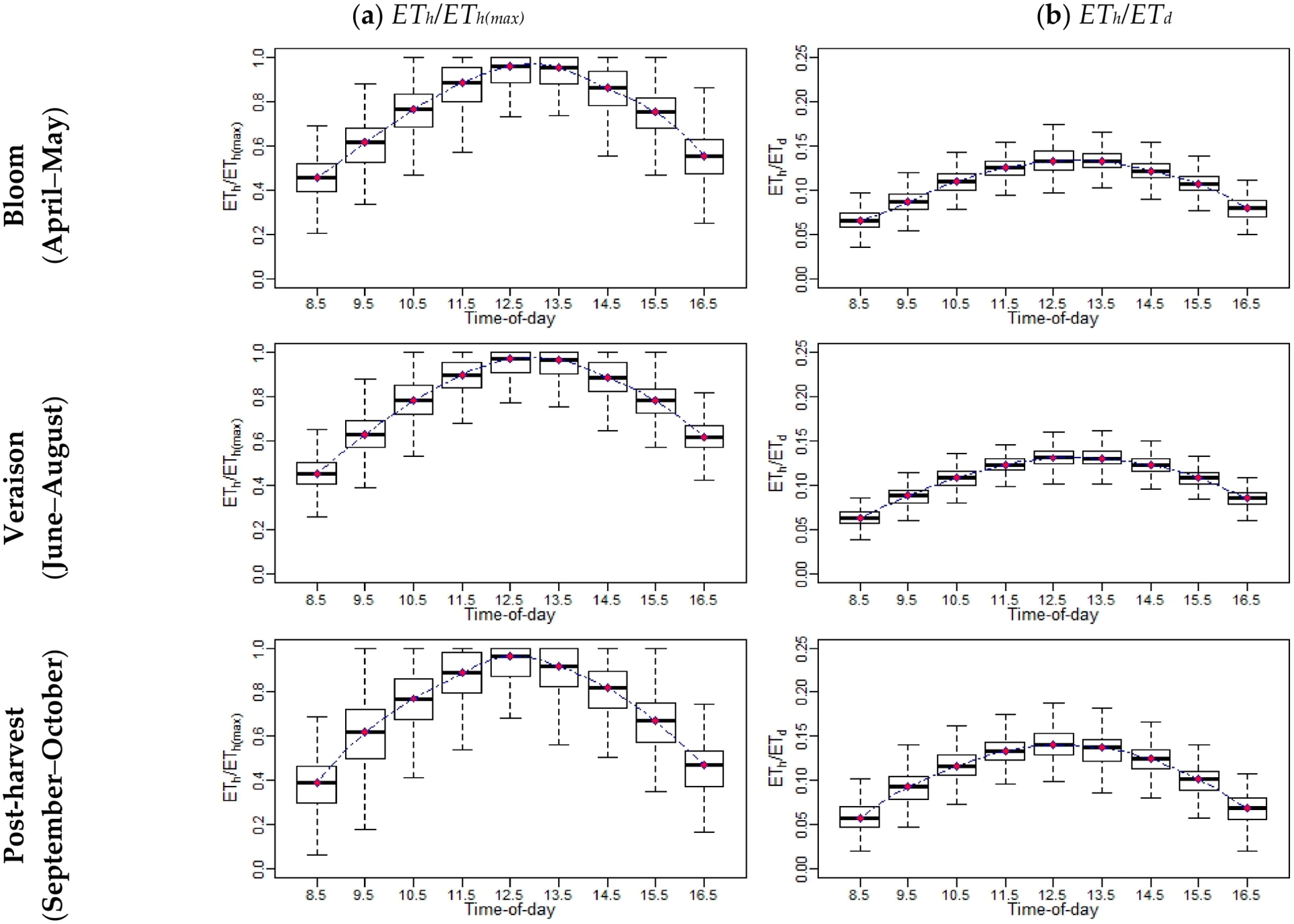
An example of the diurnal variations of (**a**) and *ET*_*h*_*/ET*_*h(max*)_ and (**b**) *ET*_*h*_*/ET*_*d*_ at different phenological vine stages for Sierra Loma Sites 1 and 2 between 2014 and 2018.

To compare the contribution of the *ET* at different hours to the daily *ET*, additional statistics were included, such as the ratio of hourly *ET (ET*_*h*_)-to-daily *ET* (*ET*_*h*_*/ET*_*d*_) and the ratio of *ET*_*h*_-to-maximum hourly *ET* (*ET*_*h(max*)_) (*ET*_*h*_*/ET*_*h(max*)_). An example of the diurnal variation of both ratios (*ET*_*h*_*/ET*_*d*_ and *ET*_*h*_*/ET*_*h(max*)_) at different phenological vine stages (bloom, April–May; veraison, June–August; and post-harvest, September–October) is shown in Figure 6 for the Sierra Loma vineyard, while the figures of other sites are shown in Appendices B.2, B.3, C.2, C.3, D.2 and D.3. The general trends of *ET*_*h*_*/ET*_*d*_ and *ET*_*h*_*/ET*_*h(max*)_ resemble a Gaussian behavior, with peak values at solar noon. The results also indicate that the vine phenological stage could affect both ratios in terms of the variation at each individual hour during the daytime. In the veraison stage, low variation was observed in the *ET*_*h*_*/ET*_*d*_ and *ET*_*h*_*/ET*_*h(max*)_ compared with the bloom and post-harvest stages. In the early growing season (April), the inter-row cover crop was at peak greenness, which was senesced by early June as the vines’ leaves were fully developed (see the phenocam data at different study sites showing the different vine phenological stages: https://hrsl.ba.ars.usda.gov/awhite/CAM/ accessed 10 December 2020). This transition resulted in the main source of transpiration from the inter-rows, where the turbulent exchange was relatively suppressed to the vines with high potential coupled with the atmosphere [67]. On the other hand, the high variability observed in *ET*_*h*_*/ET*_*d*_ and *ET*_*h*_*/ET*_*h(max*)_ ratios in the time period between September and October were due to vines senescence and stress in the post-harvest stage due to a lack of irrigation and low atmospheric demand, where the daily *ET* decreased significantly. Moreover, as shown in Figure 6, the results of the *ET*_*h*_*/ET*_*d*_ indicate that the major contribution of the daily *ET* came from the midday time between 1030 to 1530, which represents at least 65% of the daily total. However, in early morning (~0630 to 0930) and evening (~1630 to 1930), the value of *ET*_*h*_*/ET*_*d*_ was low, which together represents 25–35% of the daily *ET*.

### 3.2. Comparison between Different ET_d_ Extrapolation Approaches Using the EC Measurements

Table 3 lists the goodness-of-fit statistics comparing the five different extrapolation approaches used to compute daily *ET* from the hourly *EC* at two different time windows: near solar noon (1030–1330) and afternoon (1430–1630) PST. The detailed statistics for *RMSE* and *E*_*r*_ at each individual hour at the different vineyard sites are shown in Appendices A.1, A.2, B.4, B.5, C.4, C.5, D.4 and D.5 The analysis also considered all months segregated into three vine stages/periods (April–May, June–August, and September–October) to investigate how vine phenology could affect the accuracy of estimated daily *ET* due to the timing of both water uptake and growth. In general, the results indicate that the performance of the methods had different utility in computing an accurate daily *ET* at different vine canopy development and grapevine phenological stages (bloom, veraison, and post-harvest). As shown in Table 3, the *MAPE* was lower during the summer months (June–August) compared with the early growing season (April–May) of the vine crop and after harvesting time. Meanwhile, the results indicate that the extrapolated *EC*-derived *ET*_*d*_ could be affected by the time during the day, as a better agreement was observed using instantaneous (hourly) *EC ET* between 1030 and 1330 PST than within the second time window (1430–1630 PST). Across multiple *ET*_*d*_ upscaling approaches during the veraison stage and in the 1030–1330 time window, the *MAPE* yielded values ranging between 8% and 22%, while in the 1430–1630 time window, the *MAPE* range increased and yielded values between 15% and 35%.

The results indicate that three methods (*R*_*s*_, *GA*, and *EF*) among the five daily *ET* models have the best performance (low *RMSE* and *MAPE* values and a high *NSE* value). The *R*_*s*_ showed better agreement with the ground measurements among the other extrapolation approaches and was less sensitive to *LE* variation due to seasonal and climate differences, and particularly when using the one-time-of-day *ET* in the time window between 1030 and 1330. Using the *R*_*s*_ approach, *RMSE* values were less than 0.4 mm/day, while the *NSE* value was higher than 0.9 for all vine stages (season). These results are also supported by a previous study conducted by Cammalleri et al. [29], which compared several upscaling daily *ET* methods using observations from flux towers within the United States and were evaluated over multiple seasonal cycles. They reported that using solar radiation (*R*_*s*_) for converting the instantaneous to a daily *ET* value is more robust. Comparing the less accurate daily *ET* extrapolation techniques, the Sine method marginally outperformed the *R*_*n*_*/R*_*s*_ approach in terms of moderate to high error within the time window (1030–1330) in the bloom and veraison stages, while in the post-harvest stage, the *R*_*n*_*/R*_*s*_ method gave better results than the Sine approach. Using these approaches increased the *RMSE*, which yielded values above 0.65 mm/day, while the *MAPE* values were greater than 20% in the time window between 1030 and 1330 for all vine stages (season). This implies that the Sine and *R*_*n*_*/R*_*s*_ techniques do not work properly for a daily *ET* estimate in vineyards.

### 3.3. Assessing the Instantaneous TSEB ET versus EC Measurements

**Figure 7.**
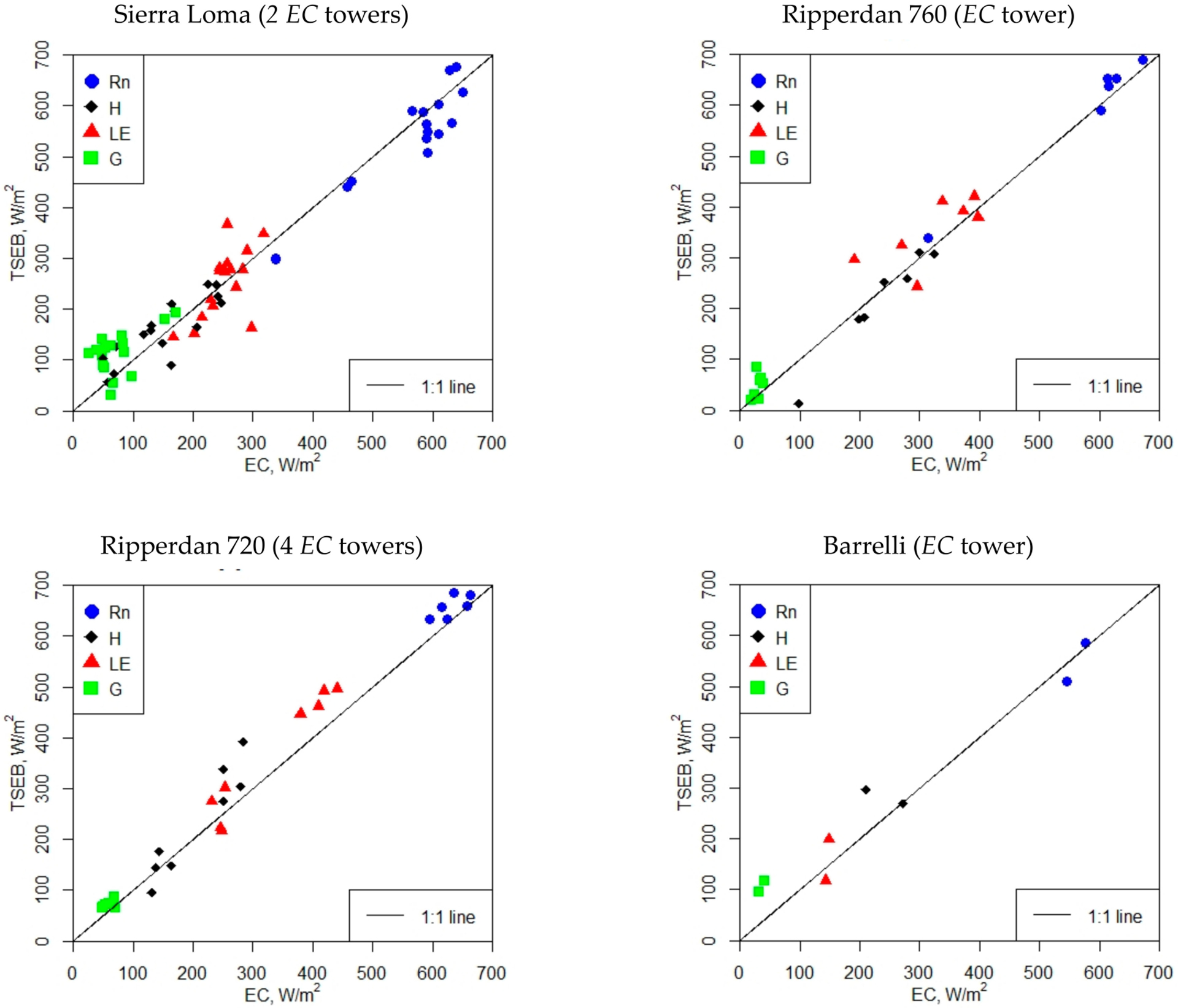
Comparison of instantaneous *TSEB sUAS* energy fluxes against *EC* measurements (without flux closure). The presented subplots include the available *sUAS* imagery, as described in Table 1.

As a first step toward evaluating the performance of the *TSEB* model, a comparison between the field observations from the *EC* and modeled fluxes using the *TSEB* and the *sUAS* (Table 1) at four different study sites are presented in Figure 7. A more detailed model performance assessment for each energy flux term is shown in Table 4. Surface fluxes were estimated from the *sUAS* based on the *TSEB* model, averaged over the *EC* footprint, and then compared against the measured fluxes. As shown in Figure 7, the estimated fluxes derived from the *TSEB* model generally align along the 1:1 line at the different vineyard sites, indicating good agreement between the modeled and measured fluxes. Net radiation (*R*_*n*_) demonstrates a close agreement with the in situ measurement, as indicated by lower *RMSE*, *MAE*, and *MAPE* values, and a high *NSE* value. The *MAE* and *MAPE* for *R*_*n*_ estimates at the different vineyard sites were less than 40 W/m^2^ and 10%, respectively, while the *RMSE* ranged between 26 W/m^2^ and 43 W/m^2^. The *NSE* yielded high values at the Sierra Loma and Ripperdan 760 sites, accounting for more than 0.85; however, at the Ripperdan 720 and Barrelli vineyards, the values decreased to less than 0.2 and 0.6, respectively. The results for *H* agreed well with the *EC* observations at the Sierra Loma and Ripperdan sites, with the *MAE* and *MAPE* values less than 43 W/m^2^ and 28%, respectively, while the *RMSE* values were less than 55 W/m^2^. However, at the Barrelli vineyard, the *RMSE* and *MAE* increased to 62 W/m^2^ and 46 W/m^2^, respectively, while the *MAPE* value was 22%. However, this site had only 2 samples to compute the difference statistics, making it difficult to reach any conclusions concerning the model performance in relation to the other sites. The results for *LE* indicate a slight increase in the *RMSE* compared to the *H*, varying between 51 W/m^2^ and 58 W/m^2^ at the Sierra Loma and Ripperdan vineyards. However, the Barrelli site results indicate that the *RMSE* of the *LE* was less than the *H*. Overall, the higher values of the *RMSE* obtained for the *LE* are attributed mainly to the *TSEB* method for calculating the *LE*, which is solved as the residual component of the surface energy balance, *LE* = *R*_*n*_-*H*-*G*. Therefore, the uncertainties associated with the calculation of energy fluxes (*R*_*n*_, *H*, and *G*) within the *TSEB* method can adversely affect the estimation of the *LE*. Another potential uncertainty could be related to the no use of flux closure in the eddy covariance (*EC*) data. According to previous studies (e.g., Neale et al. 2012) [68], heat fluxes (*H* and *LE*) are acceptable when the *RMSE* ranged between 20 W/m^2^ and 60 W/m^2^. This implies that the results of the *H* and *LE* obtained from the *TSEB* model across different vineyards were within an acceptable range and similar to prior studies [50]. The results for *G* indicate poor performance across the different vineyard sites, except for Ripperdan 720 vineyard, which had a *MAPE* of less than 25%. Part of these discrepancies between the modeled and observed *G* can be attributed to the assumption used in this study for calculating *G*, which is that as a portion of the soil net radiation (*R*_*ns*_), *G* = 0.35 *R*_*ns*_. This value was obtained based on a proposed method by Nieto et al. [49], which takes into consideration the diurnal variation of the *G/R*_*ns*_ and found high scattering/uncertainty in the relationship, with an average value of 0.35 near solar noon. In this study, most of the flights were between 1000 and 1500, and at these time intervals around solar noon, the *G/R*_*ns*_ fraction remained rather constant at ~0.35 (see Figure 4 in Nieto et al. (2019)) [49]. Therefore, for the sake of simplicity, and considering that the sinusoidal approach might be site-dependent, the constant fraction at 0.35 was used. This value is also broadly applied over a wide range of crops and environments. Meanwhile, vineyards are characterized by strong heterogeneity, which causes spatial and temporal variability in *G* values. According to Kustas et al. [69], the simple remote sensing methods for estimating *G* as a portion of *R*_*n*_ have significant uncertainty due to temporal variability in the *G/R*_*n*_ ratio.

### 3.4. Assessment of the Daily ET Extrapolation Approaches Using TSEB sUAS Results

**Figure 8.**
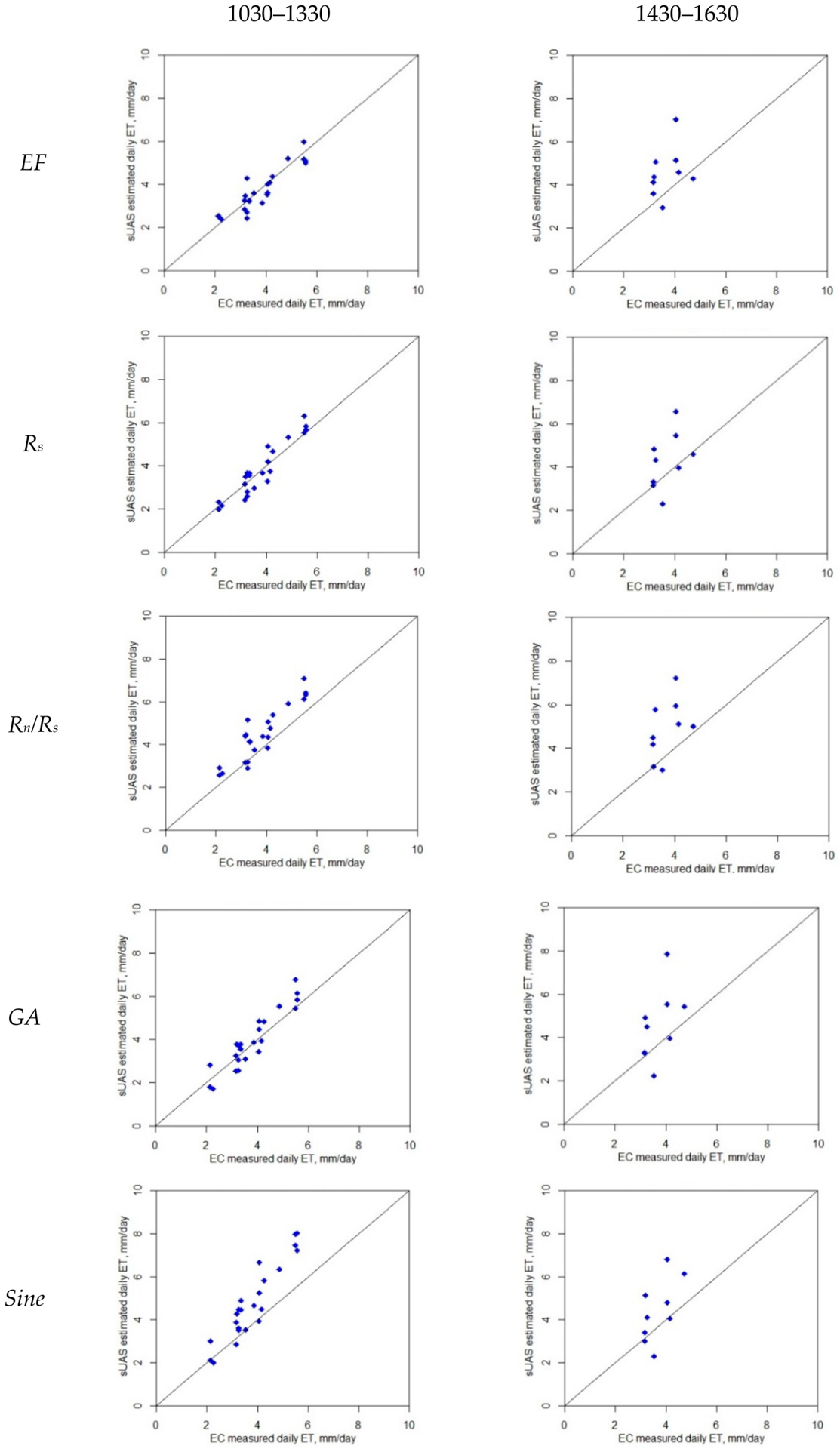
Comparison between daily *ET* from *TSEB sUAS* and *EC* at two different time windows (1030–1330 and 1430–1630).

The accuracy of the daily high-resolution *ET* from the *TSEB* depends largely on an accurate instantaneous *ET* estimate at the time of acquisition of the *sUAS* imagery, as well as the reliability of the approach used to scale up the *TSEB*-derived *ET* to a daily value. The five daily *ET* methods (*EF*, *R*_*s*_, *R*_*n*_*/R*_*s*_, *GA*, and Sine) were applied using the modeled energy fluxes derived from the *TSEB* and compared against the *EC*-derived daily value, *ET*_*d*_, calculated by integrating the daytime *LE* fluxes measured by *EC* towers. Table 5 lists the goodness-of-fit statistics between the modeled daily *ET* using *sUAS* data sets and the ground-based *EC* daily measurements at two time windows during the day: 1030–1330 and 1430–1630. Figure 8 shows the relationship between the modeled and measured fluxes. Overall, the results indicate that the modeled *ET*_*d*_ values have better agreement across different upscaling methods using the time window of 1030–1330 PST, while a significant deterioration was observed in the performance of all methods using the 1430–1630 period for upscaling. The *RMSE* and *MAPE* statistics yielded values greater than 1.2 mm/day and 25%, respectively, in the 1430–1630 time window; however, these values decreased to less than 1 mm/day and 20% across different methods using the *TSEB* output in the 1030–1330 timeframe, with one exception. In the case of the Sine approach, the *RMSE* and *MAPE* yielded values of 1.32 mm/day and 26%, respectively. These findings align with the results obtained when comparing different *ET*_*d*_ methods using measurements from the *EC* tower (see Section 3.2), where *RMSE* and *MAPE* yielded values greater than 0.5 mm/day and 14% in the time window 1430–1630. However, using the time window of 1030–1330, the values of *RMSE* and *MAPE* decreased to less than 0.7 mm/day and 23%, respectively. The larger *RMSE* and *MAPE* values obtained in the *sUAS ET*_*d*_ compared to the *EC ET*_*d*_ are due to the bias in the *TSEB*-derived *ET* compared to the *EC* measurements. These results are also supported by previous studies conducted by Jackson et al. [30] and Colaizzi et al. [28], where scaling instantaneous *ET* to daily values showed better agreement when the measurement was taken within about 1–2 h of solar noon.

Although the results indicate that three (*GA*, *EF*, and *R*_*s*_) out of the five methods for daily *ET* upscaling agree reasonably well with the ground-based measurements, the *R*_*s*_ technique yielded better agreement at all three sites (Sierra Loma, Ripperdan 720, and Barrelli). This approach generated a robust *ET*_*d*_ when a single remote sensing-based *ET* estimate was taken within 1–2 h of solar noon and provided a close agreement with the ground truth *ET* measurement. This result also aligns with the *EC ET*_*d*_ analysis, which indicates that the *R*_*s*_ approach has better statistical performance (see Table 3). Using the *R*_*s*_ approach for all vineyards, the *RMSE* values were 0.45 mm/day, and the *MAPE* was 10%, while the *R*^*2*^ was 0.88 for the time window of 1030–1330 (see Table 5, All Vineyards section).

These results agree with a previous study conducted by Wandera et al. [41], which showed that the *R*_*s*_-based approach was better for upscaling compared with the *EF* method. That study was carried out over 41 FLUXNET validation sites for two different times of day, including 1100 and 1330. Furthermore, the found results are also supported by Cammalleri et al. [29], when comparing different daily extrapolation methods. Cammalleri et al. [29] found that the incoming solar radiation (*R*_*s*_) was the most robust method with the least error when using *EC* data collected at different flux tower sites within the United States and over multiple seasons. The *R*_*s*_ approach for *ET* upscaling is highly recommended in situations where obtaining the daily net radiation is not possible [19] or, in some cases, where the modeled *R*_*n*_ is overestimated/underestimated, which will adversely affect the *EF* ratio. On the other hand, the *G* is more difficult to estimate than the *R*_*s*_ and *R*_*n*_, which could limit the accuracy of the *EF* method. This might explain why the *R*_*s*_ method has a slightly higher agreement than the *EF*. Comparing the approaches with the lowest performance, the Sine method demonstrated the worst performance, with the largest *RMSE* and *MAPE* values and the lowest *NSE* value in the time window between 1030 and 1330. However, between 1430 and 1630, the results indicate that Sine performed slightly better than *R*_*n*_*/R*_*s*_. Still, the *RMSE* and *MAPE* values were high and the *NSE* and *R*^*2*^ values were very low. The hypothesis is that the heterogeneity in the field, due to vine biomass, cover crop, and bare soil, has a larger impact on the *R*_*n*_*/R*_*s*_ and Sine approaches than other methods.

## 4. Conclusions

The objective of this study was to assess existing methodologies for upscaling *ET* from single time-of-day information to daily estimates over commercial vineyards in California’s Central Valley using *EC* flux measurements and the *TSEB* model with *sUAS* imagery. The extrapolation approaches included the evaporative fraction (*EF*), solar radiation (*R*_*s*_), net radiation to incoming solar radiation (*R*_*n*_*/R*_*s*_), the Gaussian (*GA*), and Sine technique. First, analysis was performed using flux observations collected at eight *EC* towers located at three vineyards in California’s Central Valley: Sierra Loma, Ripperdan, and Barrelli. These sites are characterized by different climates, soils, vine variety, and trellis designs. The analysis also considered months of the growing season to coincide with three vine phenological stages (April–May (rapid vine growth, bloom/berry establishment), June–August (berry development/veraison), and September–October (harvest/post-harvest/vine senescence)) to investigate how vine phenology could affect the accuracy of the modeled daily *ET* due to timing of both water uptake and growth.

The *EC* analysis results indicate that three daily *ET* approaches (*EF*, *R*_*s*_, and *GA*) out of five have a reasonable agreement with the *EC*-based measurements, with the *R*_*s*_ approach being preferred for daytime upscaling of *ET* across different stages of vine phenology, as it yielded the highest accuracy among the tested methods. Moreover, the results demonstrate that the methods could perform differently at different vine canopy development and grapevine phenology stages and at different time windows during the day. In the time window between 1030 and 1330, *MAPE* yielded values of 8% when using the *R*_*s*_ approach in the veraison stage, whereas this value increased to 17% between 1430 and 1630 h. In the bloom and post-harvest vine stages, the *MAPE* yielded values of 10% and 11%, respectively, when using *R*_*s*_ within the 1030–1330 time window, which then increased to 19% and 23%, respectively, between 1430 and 1630.

A similar result was obtained when applying the five *ET* upscaling methods using instantaneous *TSEB*-derived *ET*. The results reported that the *R*_*s*_, out of the other methods, has better agreement with the ground measurements to extrapolate the instantaneous *ET* at the time of the *sUAS* acquisition to daily values, with an *RMSE* of 0.45 mm/day and an *MAPE* of 10% in the time window between 1030 and 1330 PST. The *EF* and *GA* methods performed relatively well, with a *MAPE* of 10% and 13%, respectively, in the same time window. However, between 1430 and 1630, the results indicate a significant deterioration in the performance of all methods, with the *RMSE* and *MAPE* values greater than 1.2 mm/day and 25%, respectively. The range in climate, vine variety, soils, trellis designs, and times when *sUAS* imagery was collected support the general results that the *R*_*s*_ extrapolation method can provide reliable daily *ET* estimates, particularly if the modeled *ET* is extrapolated from imagery collected 1–2 h before/after solar noon.

## Figures and Tables

**Table 1.** Dates and times of *AggieAir sUAS* flights used in this study.

Site	Date	Time PST^1^	Spectral Bands^2^	Satellite’s Overpass
Sierra Loma	9 August 2014	1041	RGBNIR^3^	Landsat
Sierra Loma	2 June 2015	1043	RGBNIR	Landsat
Sierra Loma	2 June 2015	1407	RGBRE	NA
Sierra Loma	11 July 2015	1035	RGBNIR	Landsat
Sierra Loma	11 July 2015	1414	RGB	NA
Sierra Loma	2 May 2016	1205	REDNIR	NA
Sierra Loma	2 May 2016	1504	REDNIR	NA
Sierra Loma	3 May 2016	1248	REDNIR	NA
Barrelli	8 August 2017	1052	RGBNIR	Landsat
Barrelli	9 August 2017	1043	RGBNIR	Landsat
Ripperdan 760	24 July 2017	1035	RGBNIR	Sentinel 3
Ripperdan 760	25 July 2017	1035	RGBNIR	Landsat
Ripperdan 760	25 July 2017	1357	RGBNIR	NA
Ripperdan 760	25 July 2017	1634	RGBNIR	NA
Ripperdan 760	26 July 2017	1426	RGBNIR	NA
Ripperdan 760	5 August 2018	1044	RGBNIR	Landsat
Ripperdan 760	5 August 2018	1234	RGBNIR	NA
Ripperdan 720	5 August 2018	1044	RGBNIR	Landsat
Ripperdan 720	5 August 2018	1234	RGBNIR	NA

1 PST: Pacific Standard Time.

2 Spectral Bands explanation: R/RED = red, G = green, B = blue, RE = red edge, NIR = near infrared.

3 All *sUAS* flights included thermal information.

**Table 2.** Description of *EC* towers in vineyards that were part of this study.

Vineyard	Number of *EC* Towers	Elevation *(agl)*	*EC* Tower Name	Latitude^1^	Longitude^1^	Period of Data (Years)
Sierra Loma	2	5	1	38°16′49.76″	−121°7′3.35″	5
			2	38°17′21.62″	−121°7′3.95″	5
Ripperdan 760	1	3.5	1	36°50′20.52″	−120°12′36.60″	2
Ripperdan 720	4	3.5	1	36° 50′57.27″	−120°10′26.50″	1
			2	36°50′51.40″	−120°10′26.69″	1
			3	36°50′57.26″	−120° 10′33.83″	1
			4	36°50′51.39″	−120°10′34.02″	1
Barrelli	1	3.5	1	38°45′4.91″	−122°58′28.77″	2

1 coordinates are in WGS1984.

**Table 3.** Goodness-of-fit statistics of daily *ET* extrapolation methods at two different time windows (1030–1330 and 1430–1630 PST) using only *EC* tower information in California.

Vine Stage	Method	1030–1330					1430–1630				
		RMSE (mm/day)	MAE (mm/day)	MAPE (%)	NSE	R^2^	RMSE (mm/day)	MAE (mm/day)	MAPE (%)	NSE	R^2^
Bloom (April-May)	*EF*	0.36	0.28	10	0.83	0.85	1.02	0.71	29	−0.75	0.55
	*R*_*s*_	**0.35**	**0.26**	**10**	**0.85**	**0.87**	0.64	0.50	19	0.31	0.81
	*R*_*n*_ */R*_*s*_	1.33	0.82	29	−1.25	0.15	1.49	1.13	43	−2.68	0.06
	*GA*	0.38	0.30	11	0.81	0.87	0.87	0.72	28	−0.26	0.77
	*Sine*	0.56	0.47	18	0.60	0.86	**0.50**	**0.39**	**15**	**0.59**	**0.82**
Veraison (June-August)	*EF*	0.47	0.32	9	0.81	0.85	0.97	0.70	21	0.07	0.63
	*R*_*s*_	**0.38**	**0.29**	**8**	**0.88**	0.89	0.70	0.57	17	0.51	0.83
	*R*_*n*_ */R*_*s*_	1.67	0.90	22	−1.41	0.17	1.78	1.26	35	−2.14	0.08
	*GA*	0.43	0.33	9	0.84	0.87	1.12	0.96	29	−0.23	0.72
	*Sine*	0.65	0.53	14	0.64	0.86	**0.63**	**0.51**	**15**	**0.61**	**0.84**
Post-harvest (September-October)	*EF*	0.28	0.21	13	0.93	0.95	2.53	0.68	55	−6.76	0.10
	*R*_*s*_	**0.25**	**0.19**	**11**	**0.94**	0.95	0.49	0.37	23	0.71	0.92
	*R*_*n*_ */R*_*s*_	0.47	0.31	16	0.80	0.88	1.02	0.63	42	−0.27	0.62
	*GA*	0.40	0.31	17	0.86	0.95	0.53	0.41	25	0.66	0.93
	*Sine*	0.77	0.64	36	0.45	0.92	**0.31**	**0.24**	**16**	**0.88**	**0.92**
All stages (Season)	*EF*	0.41	0.29	10	0.91	0.92	1.50	0.70	31	−0.57	0.43
	*R*_*s*_	**0.34**	**0.26**	**9**	**0.93**	**0.94**	0.64	0.51	19	0.71	0.90
	*R*_*n*_ */R*_*s*_	1.38	0.73	22	−0.08	0.37	1.56	1.08	38	−0.71	0.23
	*GA*	0.41	0.32	12	0.90	0.93	0.95	0.77	28	0.37	0.86
	*Sine*	0.67	0.55	21	0.75	0.91	**0.54**	**0.42**	**15**	**0.80**	**0.91**

Numbers in bold are the best statistical results for each timeframe and vine stage.

**Table 4.** Goodness-of-fit statistics between the eddy covariance (*EC*) and the instantaneous *TSEB sUAS* fluxes at the different vineyard sites of this project.

Site	Fluxes	RMSE (W/m^2^)	MAE (W/m^2^)	MAPE (%)	NSE	R^2^
Sierra Loma	*R*_*n*_	43	36	7	0.85	0.90
	*H*	37	31	27	0.61	0.70
	*LE*	51	38	15	0.40	0.40
	*G*	55	50	96	0.08	0.30
Ripperdan 760	*R*_*n*_	36	31	5	0.91	0.96
	*H*	37	27	19	0.86	0.96
	*LE*	58	50	19	0.28	0.52
	*G*	27	20	66	0.11	0.21
Ripperdan 720	*R*_*n*_	35	28	4	0.17	0.53
	*H*	54	42	20	0.73	0.90
	*LE*	52	49	15	0.81	0.94
	*G*	14	14	23	−0.01	0.31
Barrelli	*R*_*n*_	26	23	4	0.58	NA^1^
	*H*	62	46	22	−0.92	NA
	*LE*	40	38	26	0.11	NA
	*G*	71	71	196	0.01	NA
All vineyards	*R*_*n*_	39	32	6	0.90	0.90
	*H*	43	34	23	0.80	0.80
	*LE*	52	43	17	0.70	0.80
	*G*	45	36	78	0.20	0.40

1 NA because we had only two *sUAS* flights.

**Table 5.** Goodness-of-fit statistics comparing multiple daily *ET* methods at two different time windows (1030–1330 and 1430–1630).

Sites	Method	1030–1330					1430–1630				
		RMSE (mm/day)	MAE (mm/day)	MAPE (%)	NSE	R^2^	RMSE (mm/day)	MAE (mm/day)	MAPE (%)	NSE	R^2^
Sierra Loma	*EF*	0.44	0.32	10	0.57	0.63	1.02	0.89	27	−7	0.00
	*R_s_*	**0.38**	**0.32**	**10**	**0.67**	**0.78**	**0.95**	**0.72**	**22**	−**6**	**0.00**
	*R_n_/R_s_*	0.95	0.77	23	−0.96	0.67	1.30	1.05	31	−12.08	0.05
	*GA*	0.44	0.39	13	0.58	0.82	1.02	0.79	24	−7.02	0.01
	*Sine*	0.80	0.63	18	−0.41	0.79	1.01	0.76	24	−6.93	0.00
Ripperdan 760	*EF*	**0.39**	**0.34**	**8**	**0.24**	**0.93**	1.85	1.5	36	−33.52	0.55
	*R_s_*	0.62	0.55	13	−0.82	0.45	**1.65**	**1.34**	**33**	−**26.54**	**0.69**
	*R_n_/R_s_*	0.73	0.62	14	−3.43	0.70	2.12	1.77	43	−44.70	0.67
	*GA*	0.63	0.61	14	−2.26	0.55	2.39	1.99	48	−56.82	0.28
	*Sine*	1.60	1.34	31	−20.18	0.19	1.83	1.63	38	−33	0.04
Ripperdan 720	*EF*	0.49	0.44	11	0.80	0.92	No flights				
	*R_s_*	**0.44**	**0.36**	**9**	**0.85**	**0.93**					
	*R_n_/R_s_*	0.83	0.73	16	0.44	0.92					
	*GA*	0.59	0.47	11	0.72	0.91					
	*Sine*	1.68	1.47	31	−1.26	0.94					
Barrelli	*EF*	0.41	0.41	19	NA	NA^1^					
	*R_s_*	**0.19**	**0.19**	**9**	**NA**	**NA**					
	*R_n_/R_s_*	0.78	0.78	36	NA	NA					
	*GA*	0.67	0.67	31	NA	NA					
	*Sine*	0.86	0.86	40	NA	NA					
All vineyards	*EF*	0.45	0.37	10	0.81	0.82	1.35	1.1	30	−14.29	0.11
	*R_s_*	**0.45**	**0.37**	**10**	**0.80**	**0.88**	**1.23**	**0.93**	**25**	−**11.65**	**0.19**
	*R_n_/R_s_*	0.87	0.73	20	0.29	0.82	1.62	1.29	35	−21.06	0.22
	*GA*	0.54	0.47	13	0.71	0.87	1.61	1.19	32	−20.72	0.25
	*Sine*	1.32	1.05	26	−0.68	0.87	1.34	1.05	28	−14.10	0.37

1 NA because we have only two observations. Numbers in bold are the best statistical results for each timeframe and vine stage.

**Figure A1.**
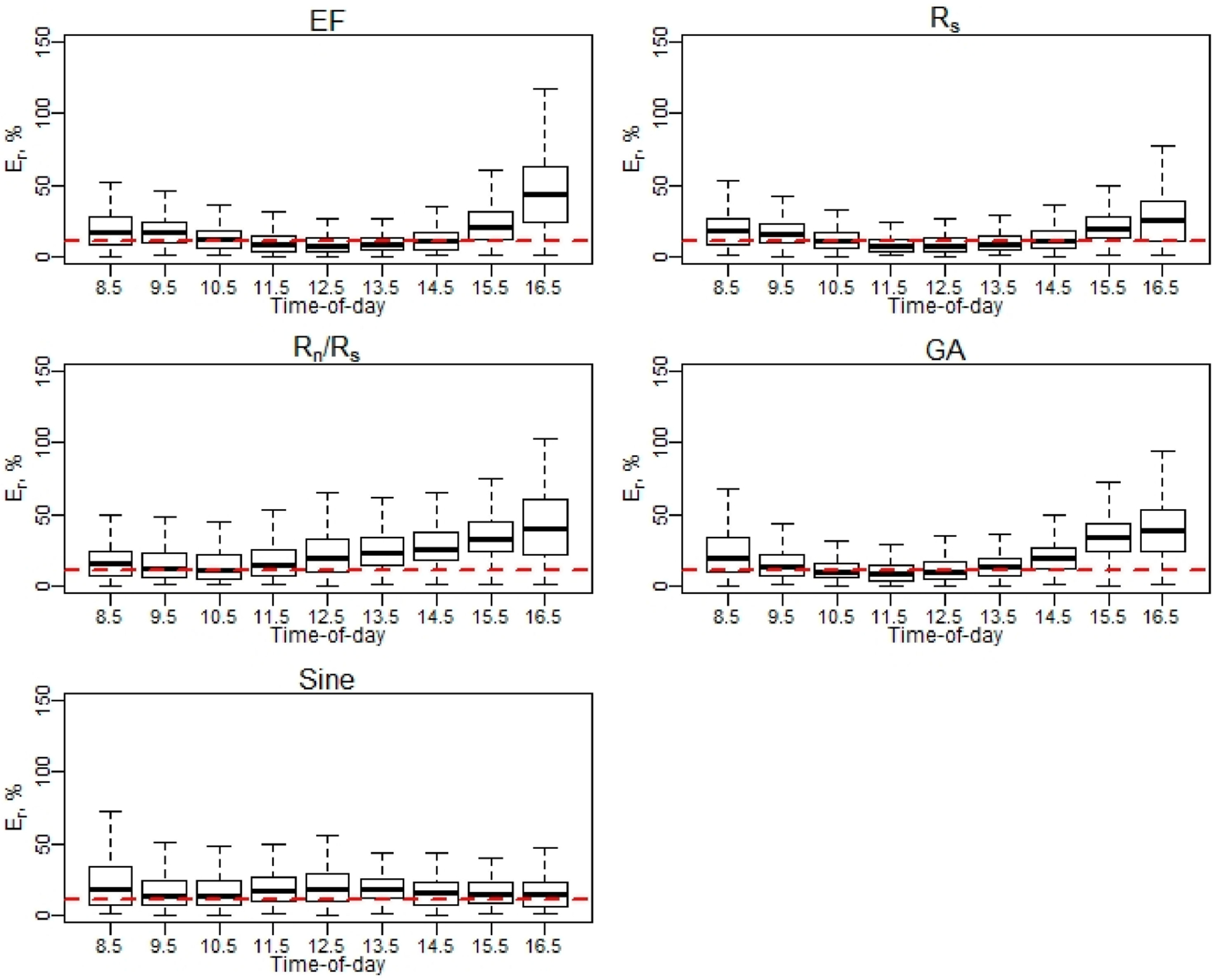
*E*_*r*_ of daily *EC ET* (April–May).

**Figure A2.**
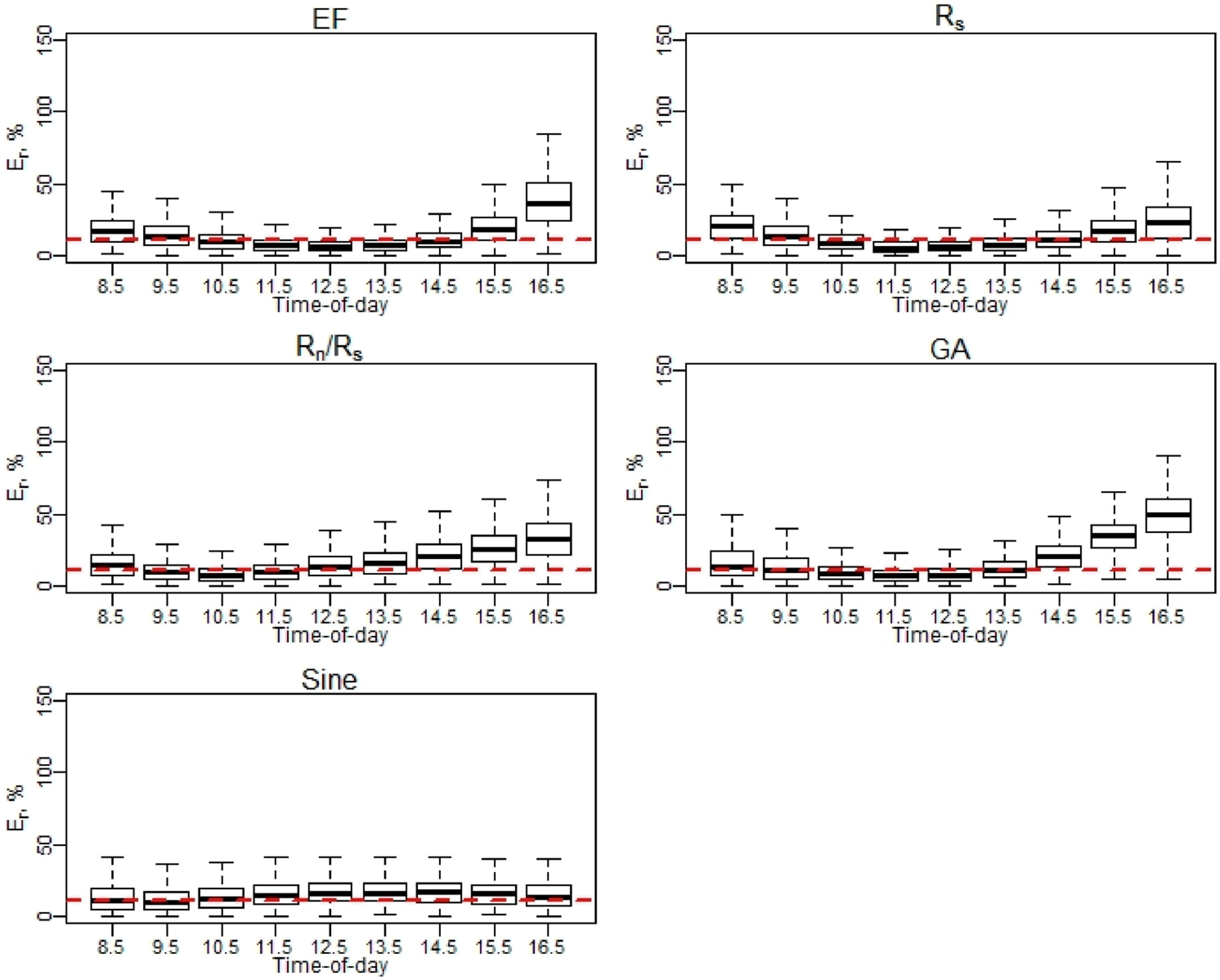
*E*_*r*_ of daily *EC ET* (June–August).

**Figure A3.**
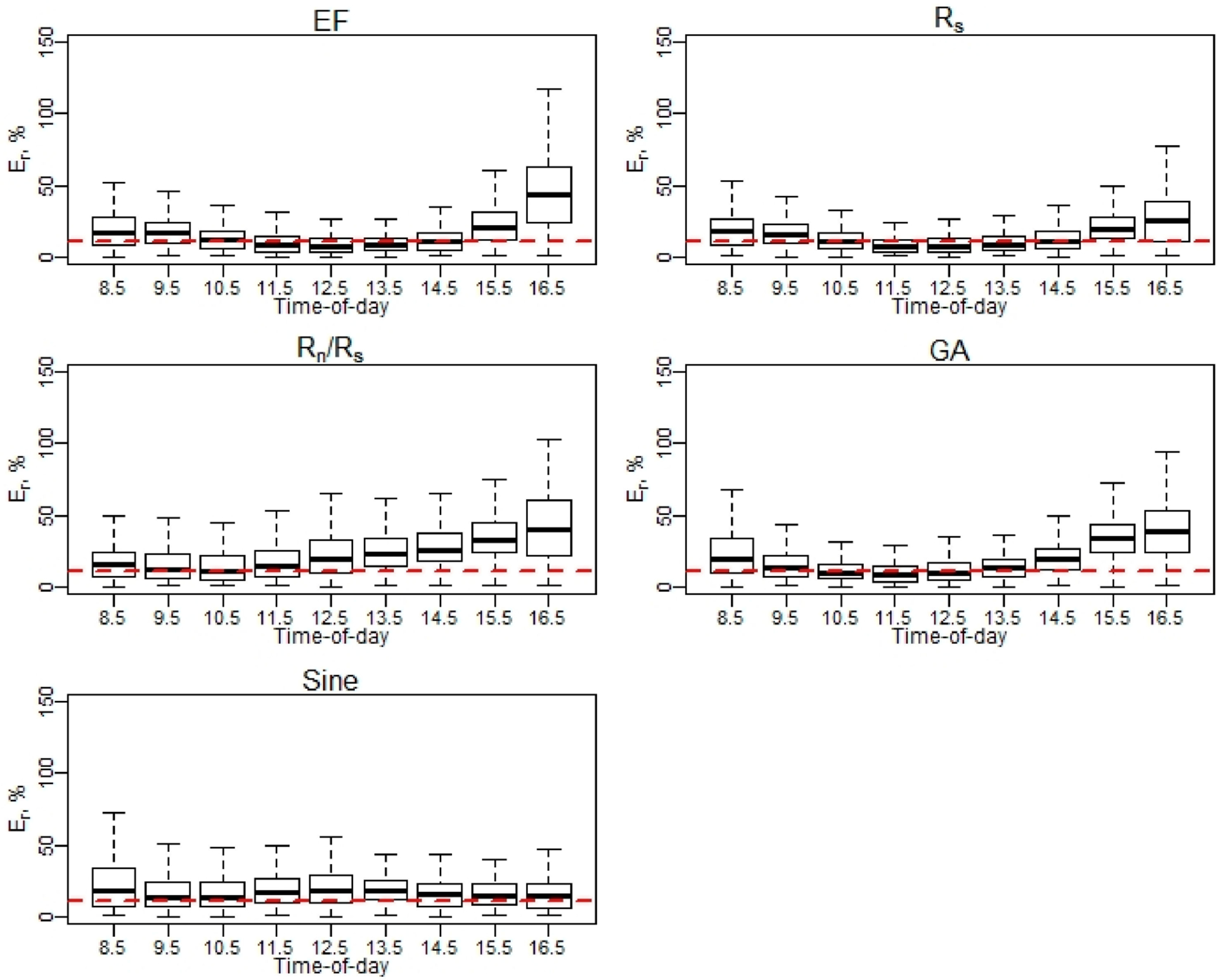
*E*_*r*_ of daily *EC ET* (September–October). Note: Red dash line represents a 10% relative error (*E*_*r*_).

**Figure A4.**
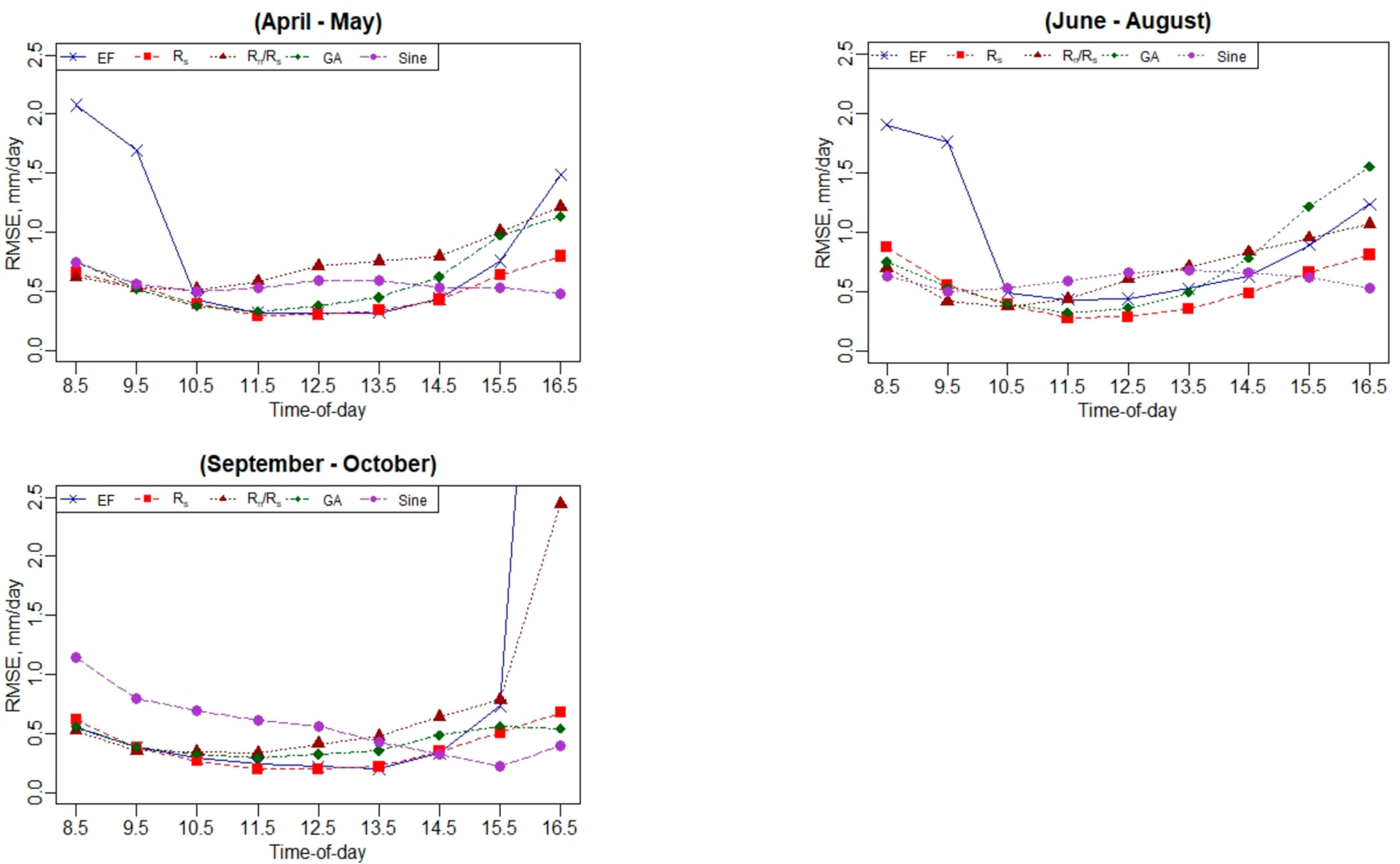
Daily *RMSE* performance using hourly *EC ET* values.

**Figure A5.**
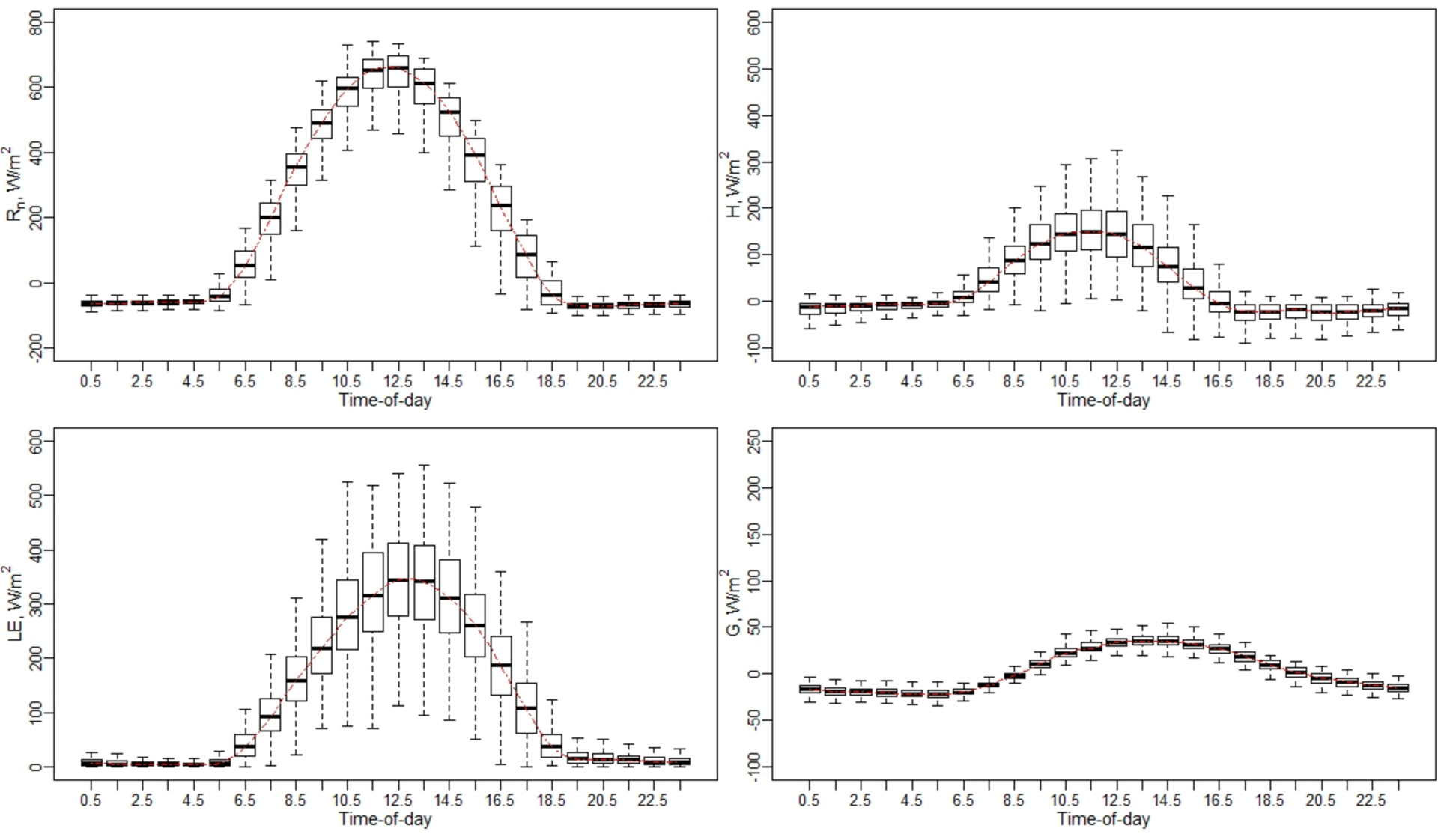
Diurnal variation of surface energy fluxes (*R*_*n*_, *H*, *LE*, and *G*).

**Figure A6.**
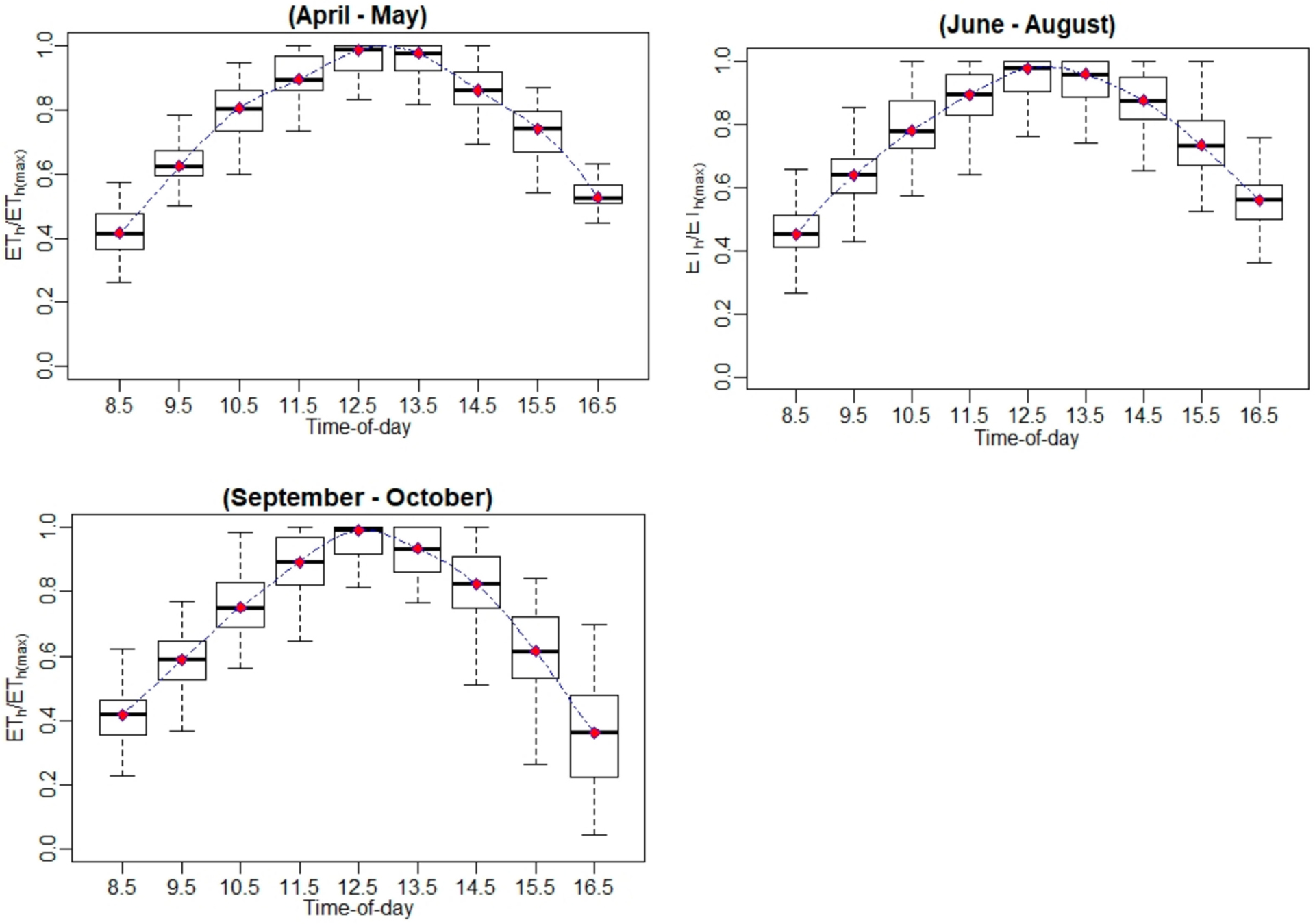
Hourly *ET*-to-maximum hourly *ET* ratio (*ET*_*h*_*/ET*_*h(max)*_) variation using *EC* measurements.

**Figure A7.**
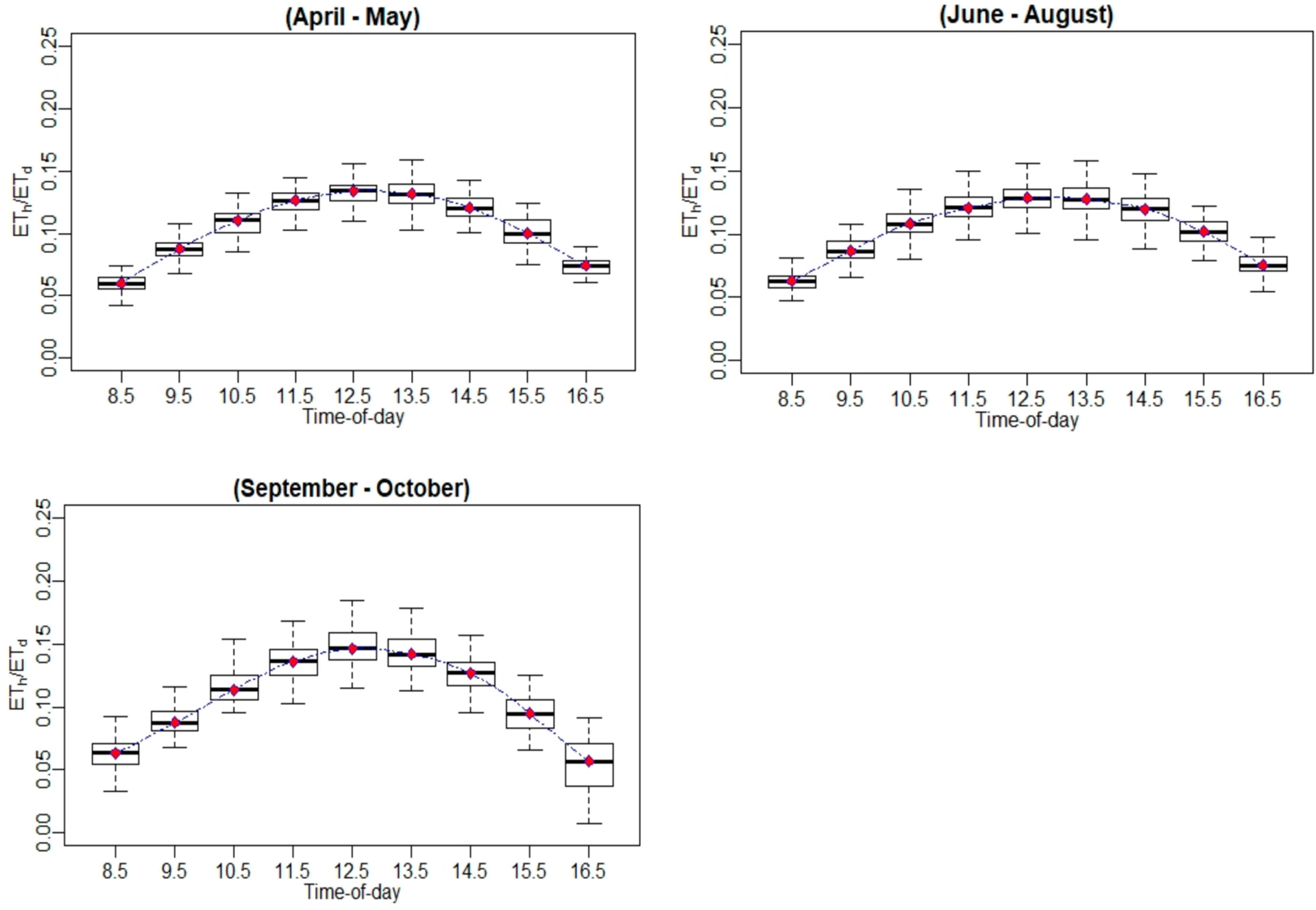
Hourly *ET* to daily *ET* ratio (*ET*_*h*_*/ET*_*d*_) variation using *EC* measurements.

**Figure A8.**
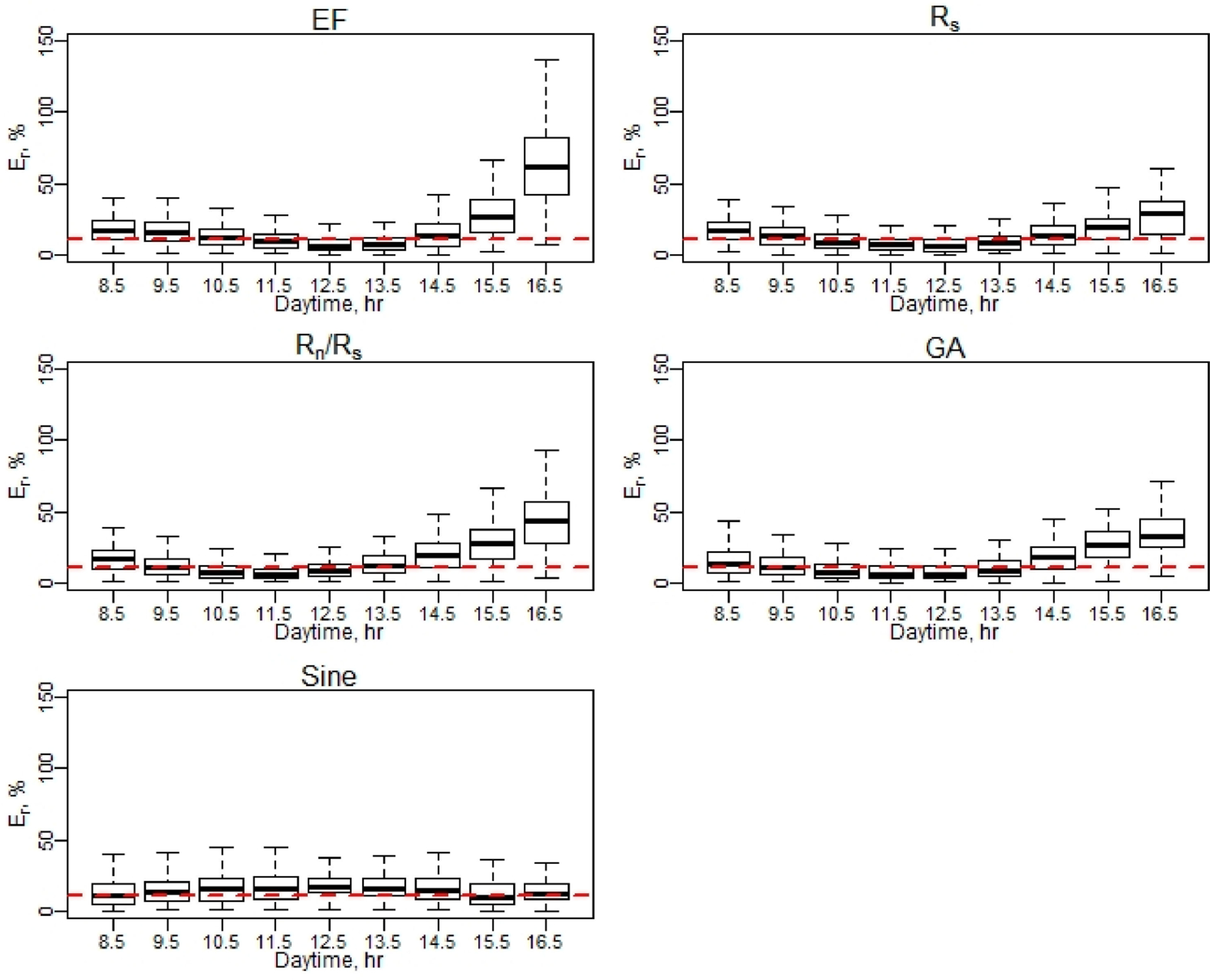
*E*_*r*_ of daily *EC ET* (April–May).

**Figure A9.**
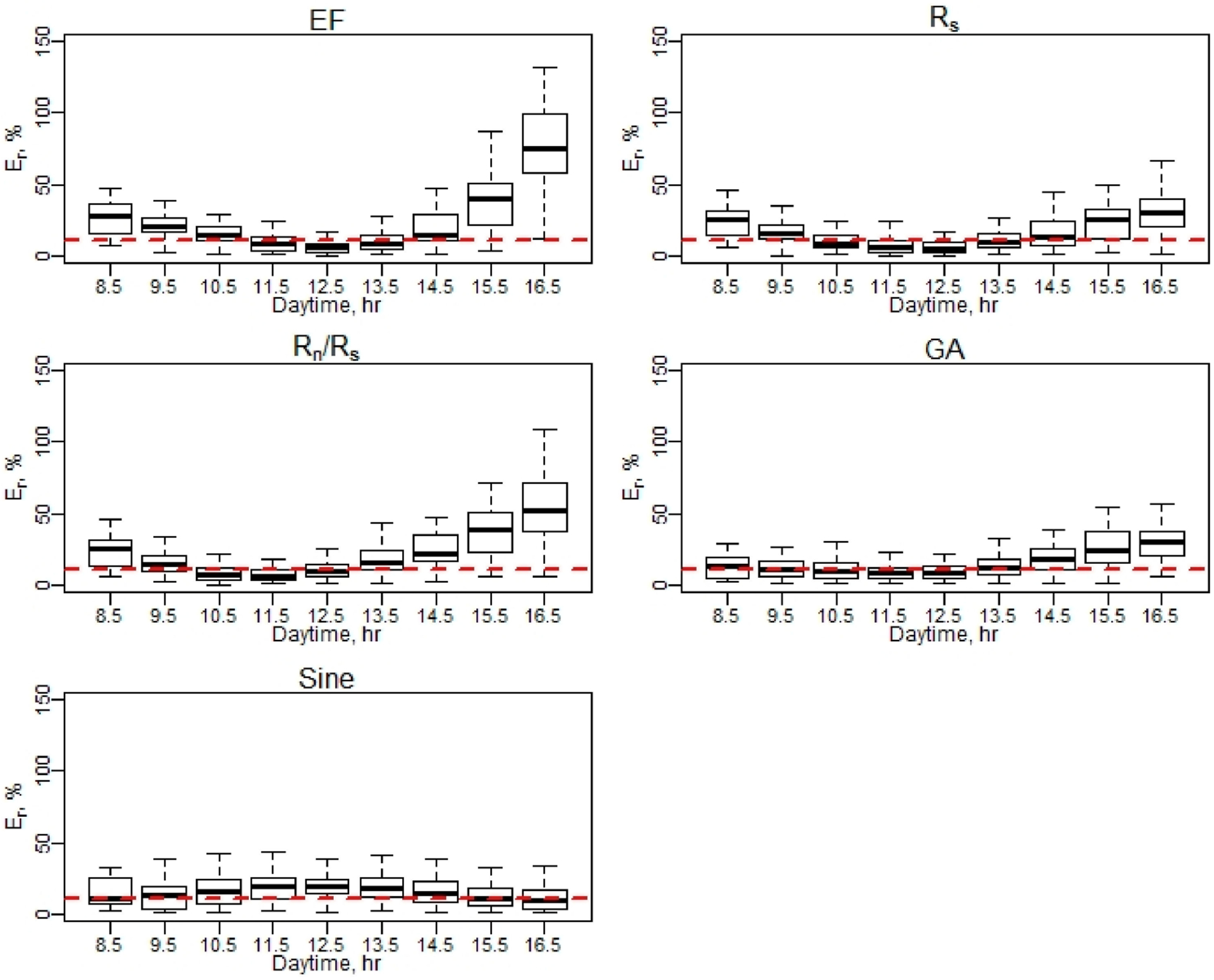
*E*_*r*_ of daily *EC ET* (June–August).

**Figure A10.**
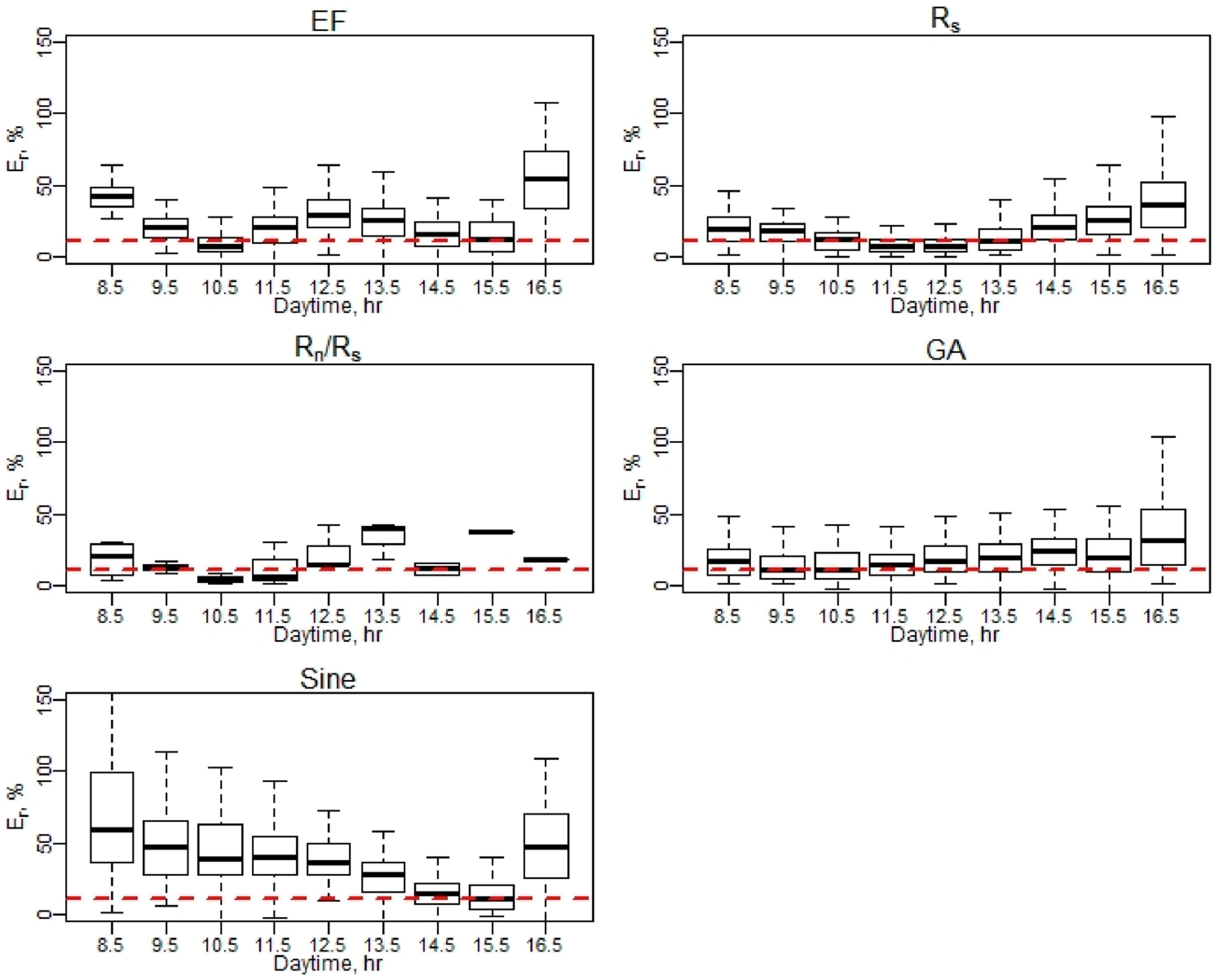
*E*_*r*_ of daily *EC ET* (September–October). Note: Red dash line represents a 10% relative error (*E*_*r*_).

**Figure A11.**
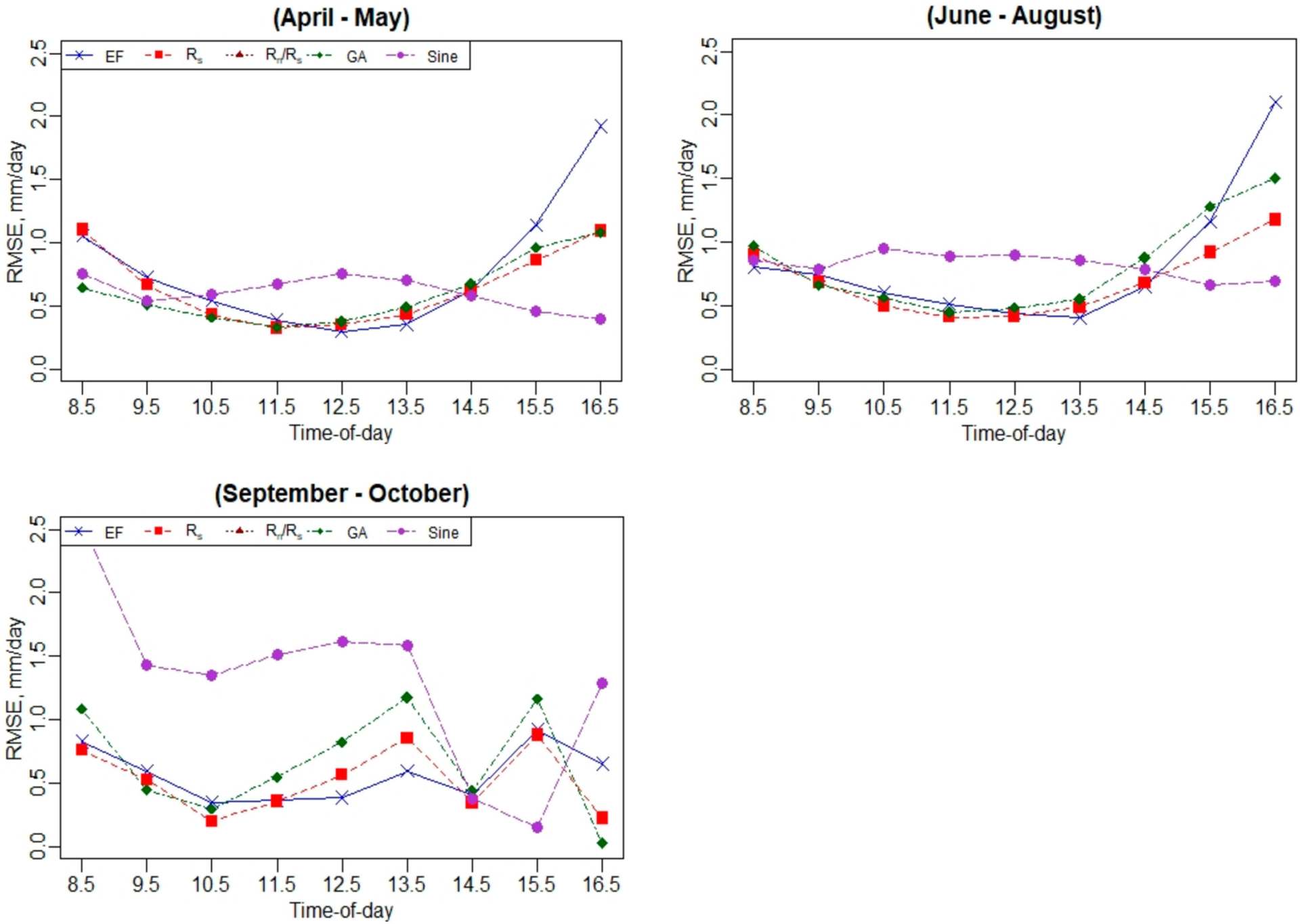
Daily *RMSE* performance using hourly *EC ET* values.

**Figure A12.**
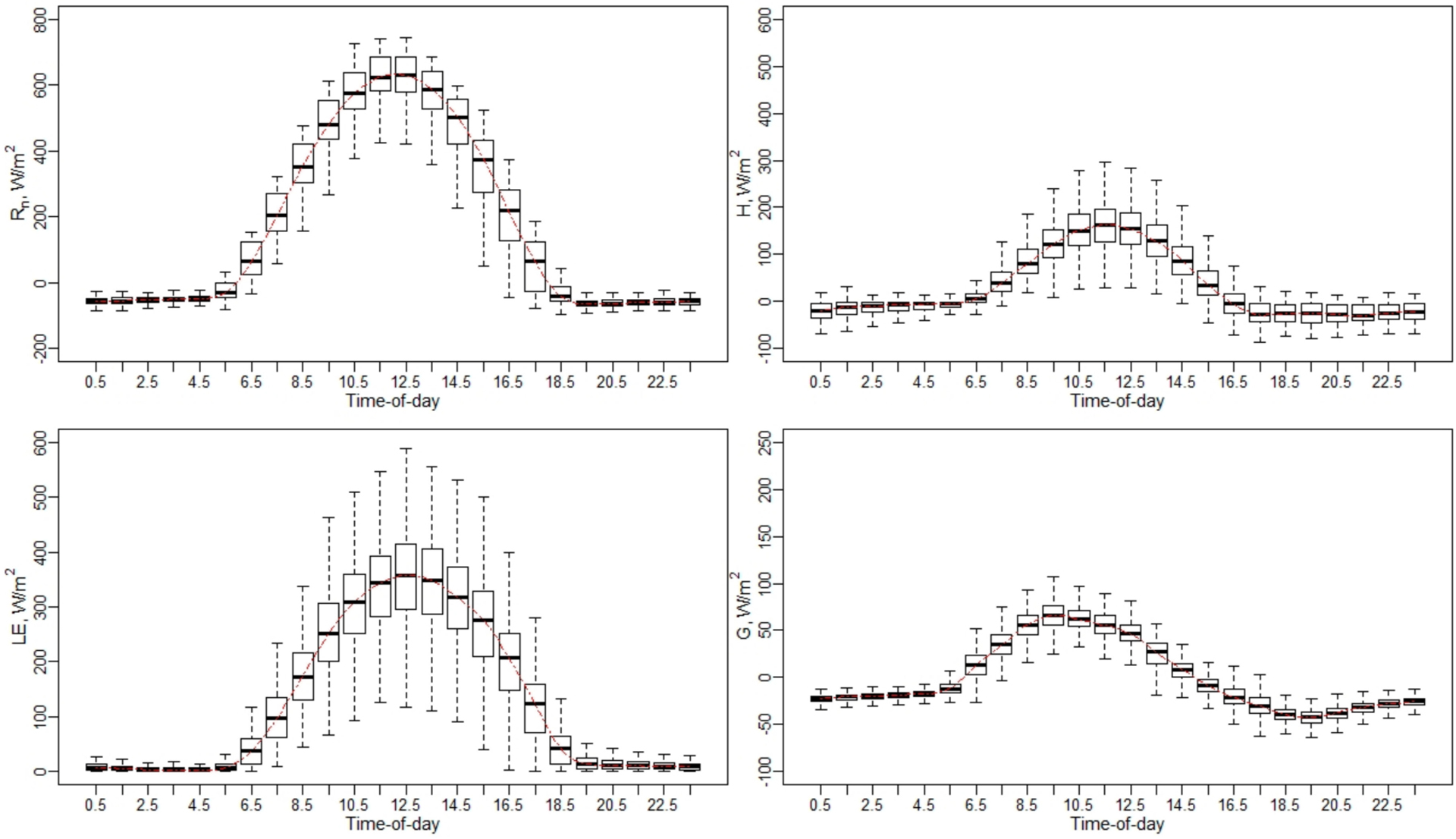
Diurnal variation of surface energy fluxes (*R*_*n*_, *H*, *LE*, and *G*).

**Figure A13.**
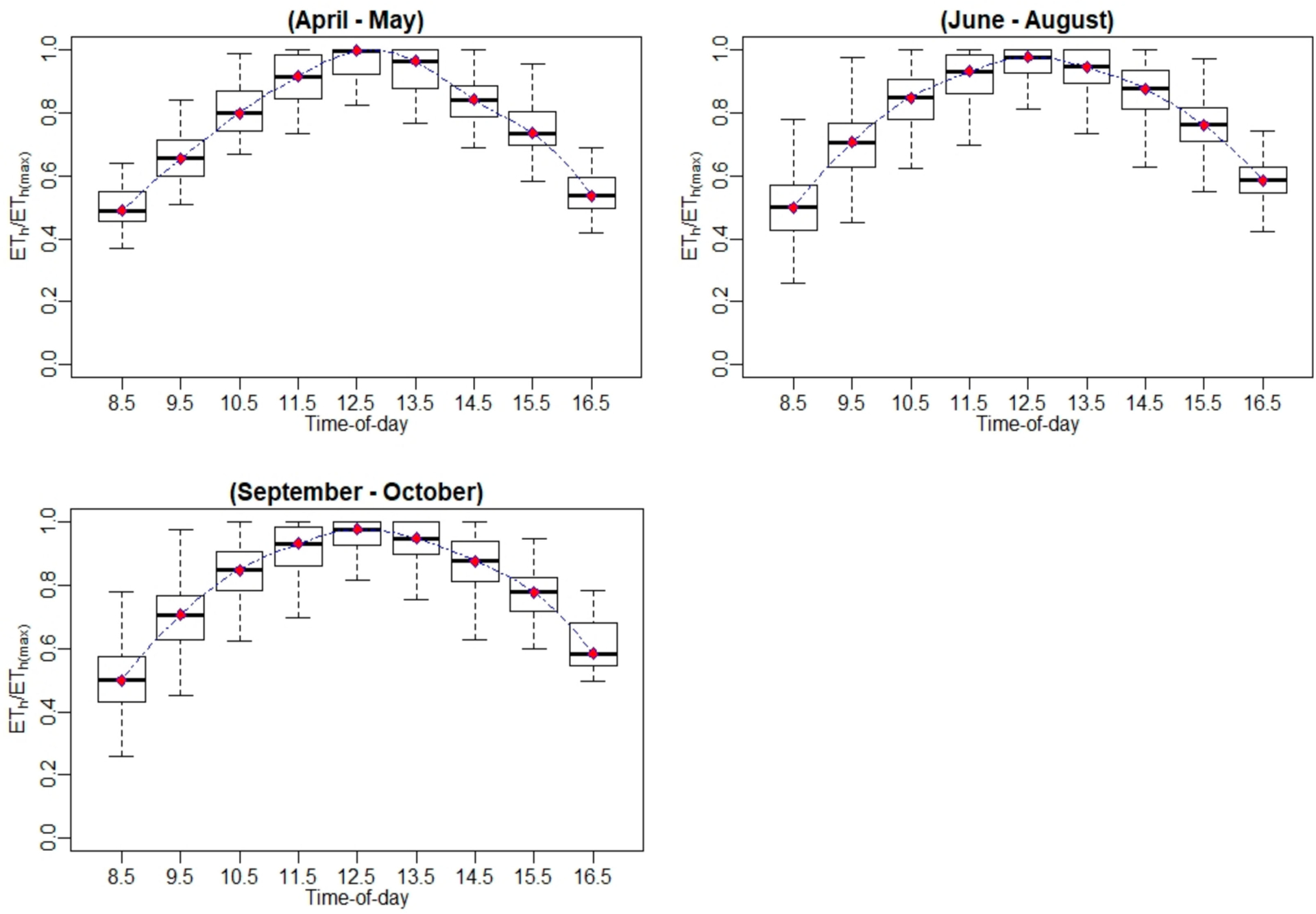
Hourly *ET*-to-maximum hourly *ET* ratio (*ET*_*h*_*/ET*_*h(max)*_) variation using *EC* measurements.

**Figure A14.**
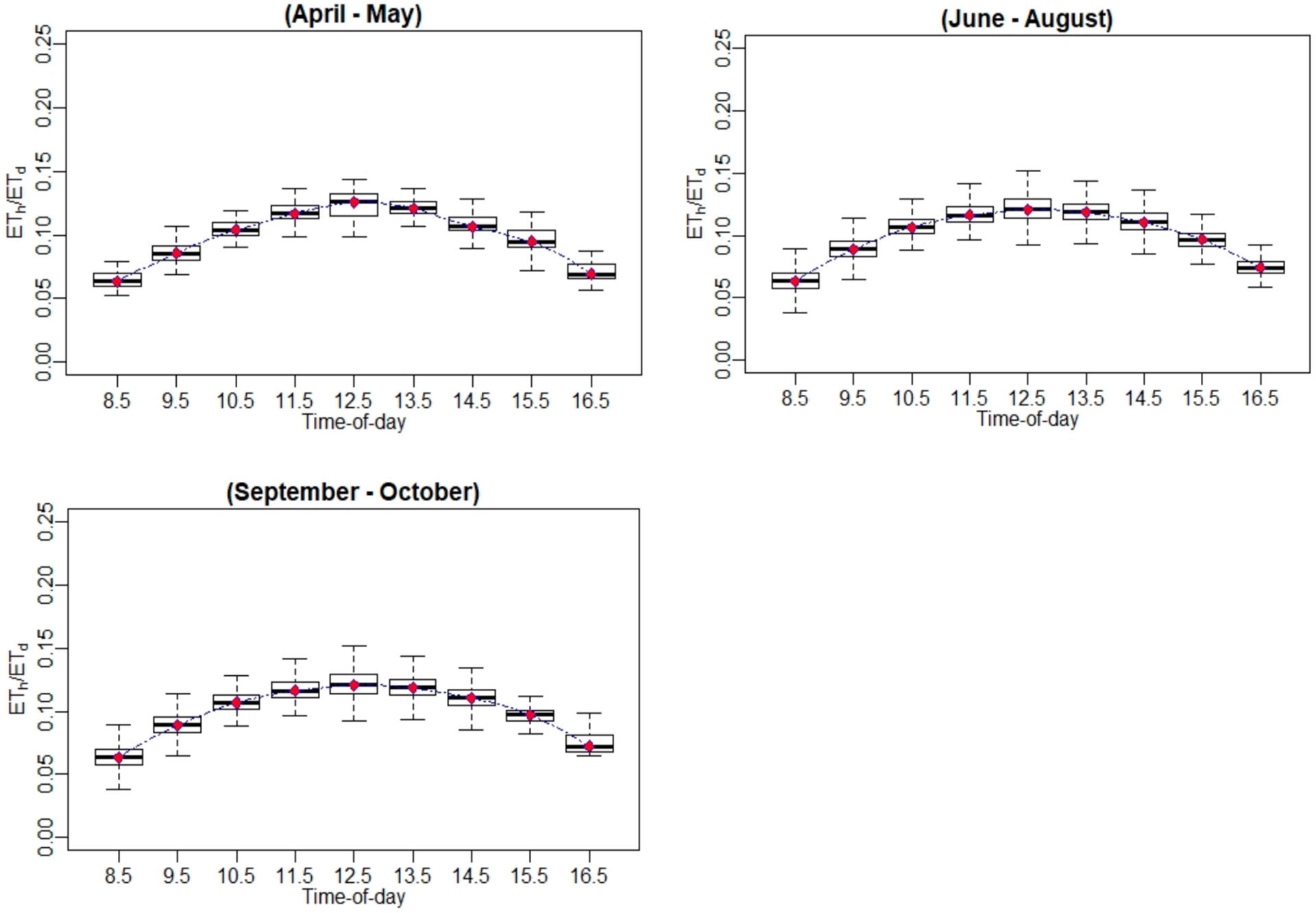
Hourly *ET*-to-daily *ET* ratio (*ET*_*h*_*/ET*_*d*_) variation using *EC* measurements.

**Figure A15.**
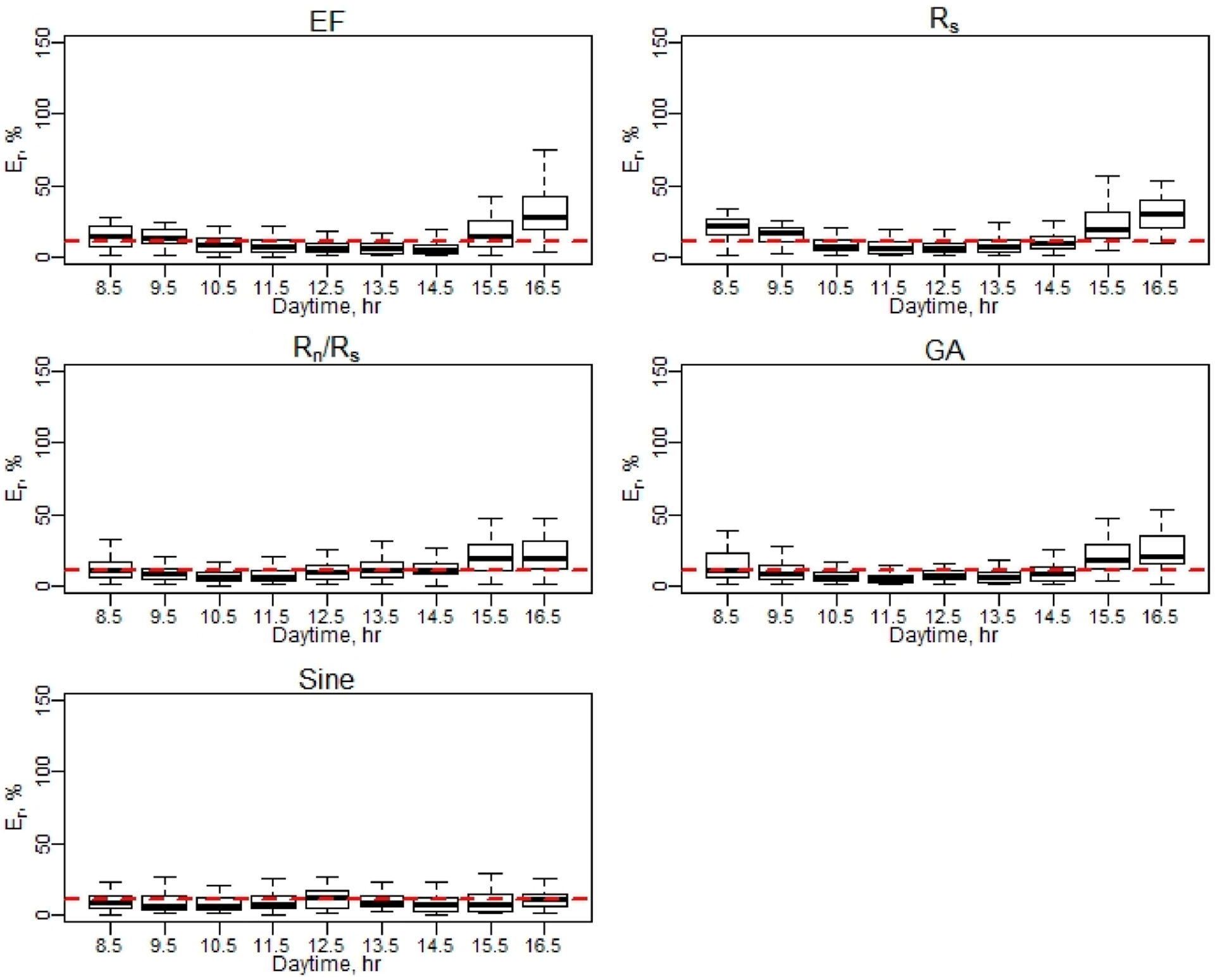
*E*_*r*_ of daily *EC ET* (April–May).

**Figure A16.**
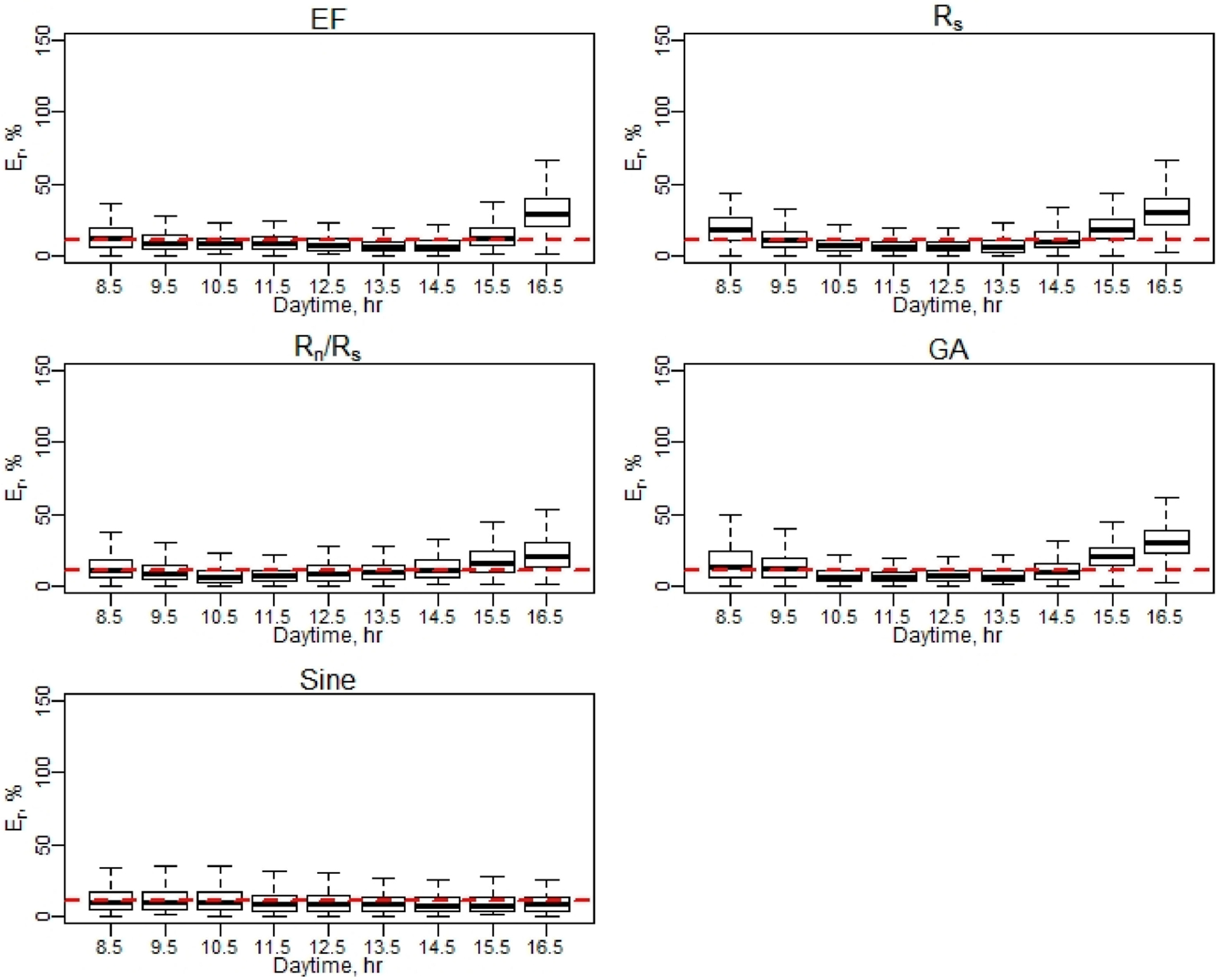
*E*_*r*_ of daily *EC ET* (June–August).

**Figure A17.**
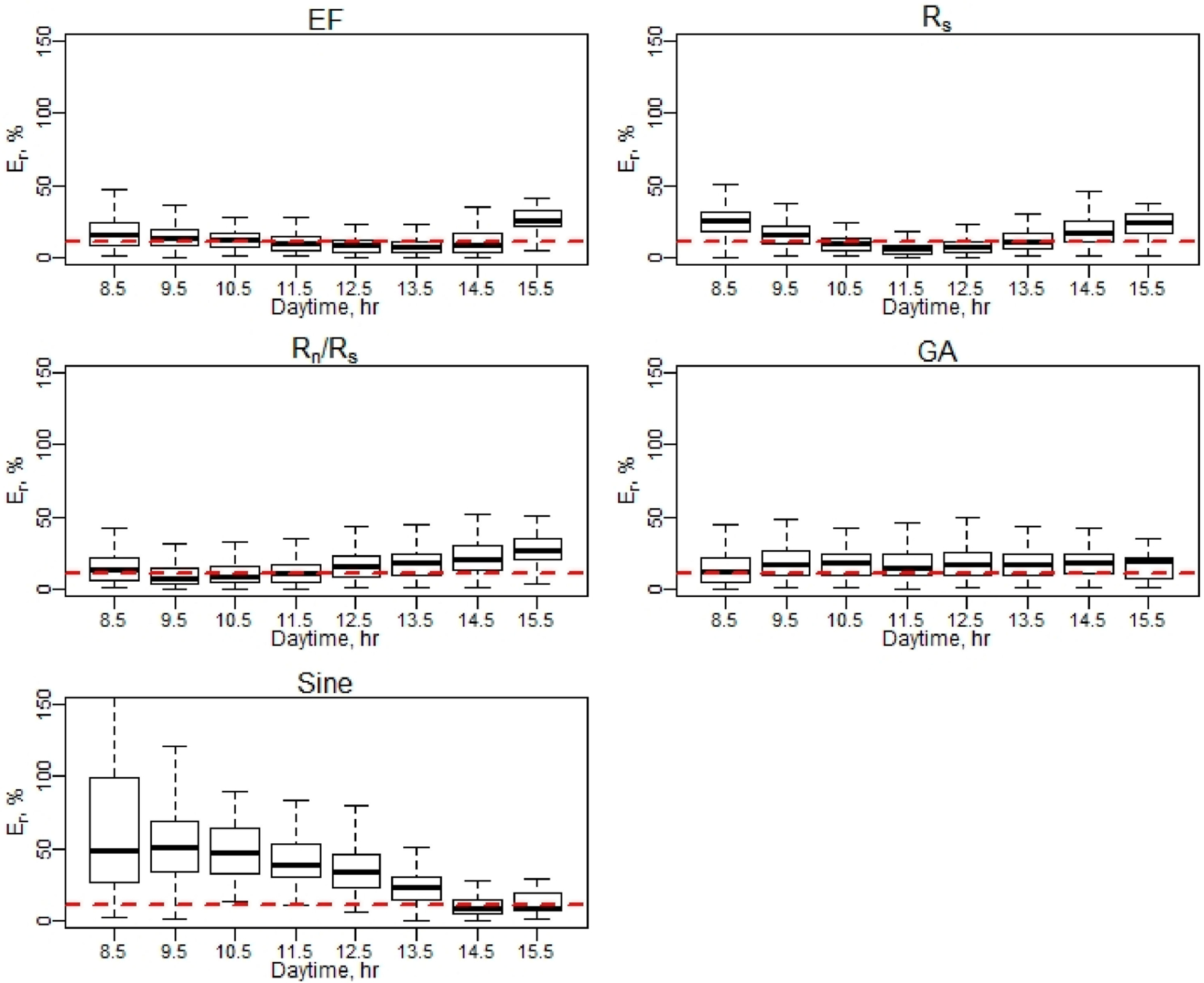
*E*_*r*_ of daily *EC ET* (September–October). Note: Red dash line represents a 10% relative error (*E*_*r*_).

**Figure A18.**
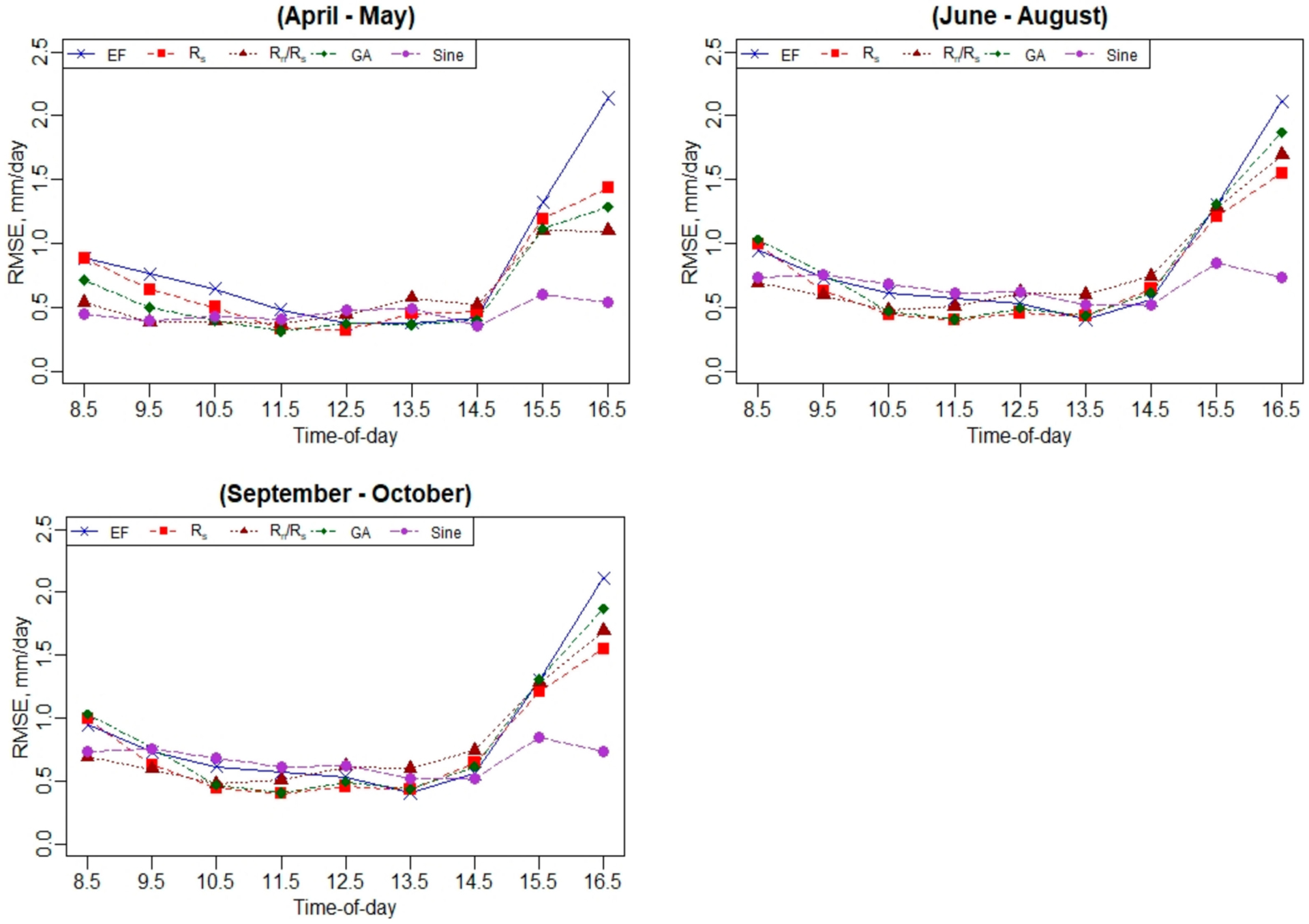
Daily *RMSE* performance using hourly *EC ET* values.

**Figure A19.**
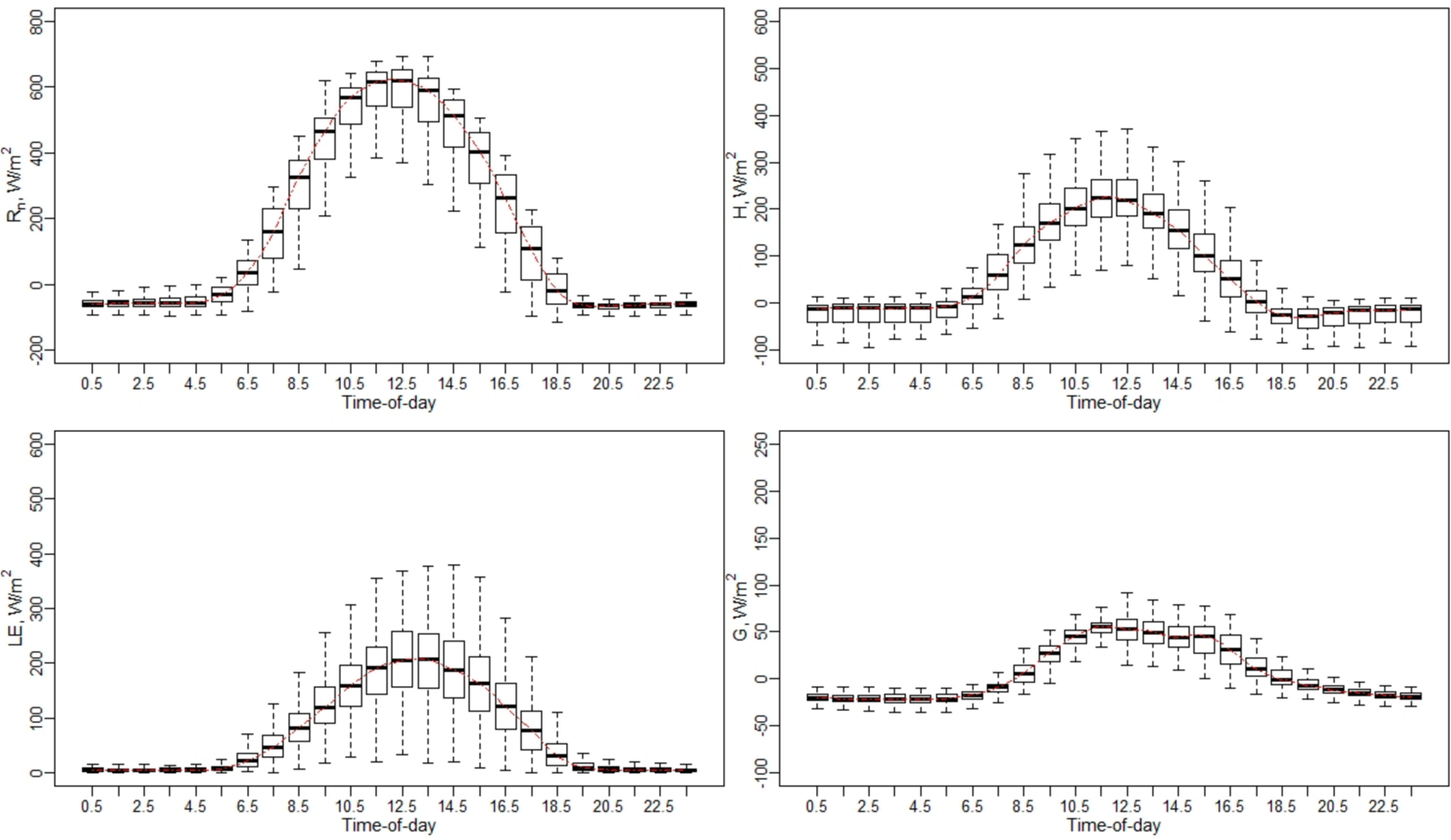
Diurnal variation of surface energy fluxes (*R*_*n*_, *H*, *LE*, and *G*).

**Figure A20.**
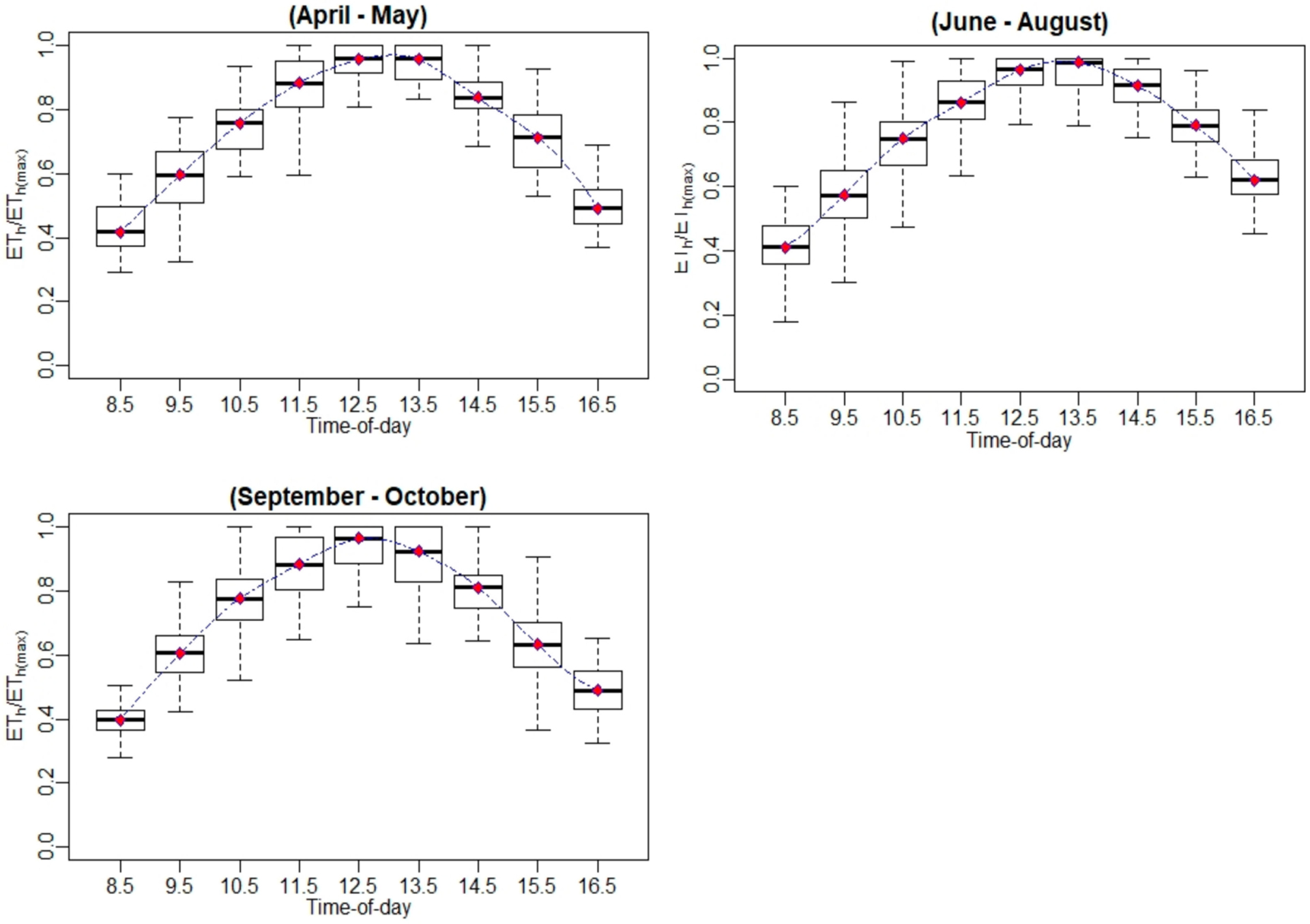
Hourly *ET*-to-maximum hourly *ET* ratio (*ET*_*h*_*/ET*_*h(max)*_) variation using *EC* measurements.

**Figure A21.**
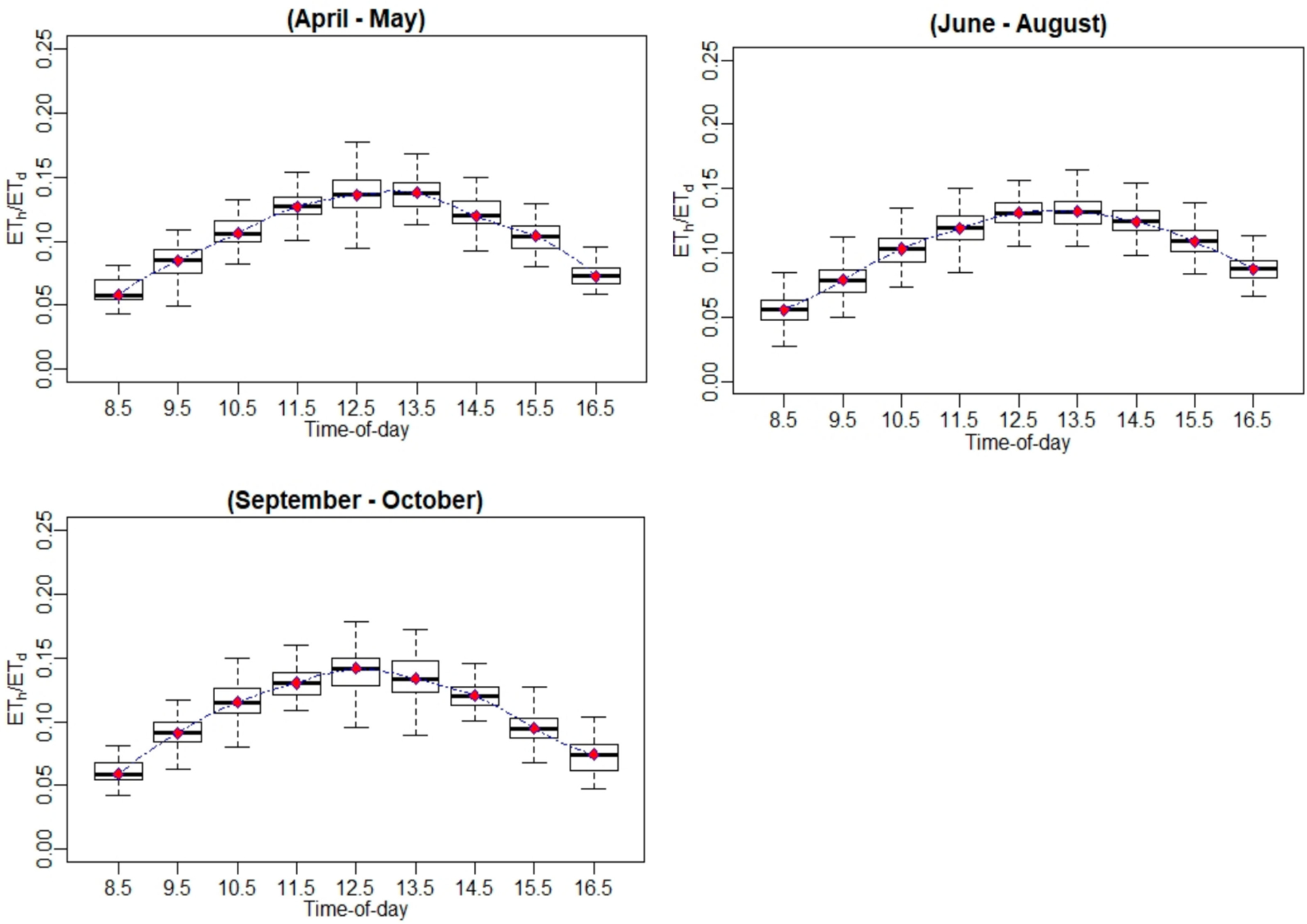
Hourly *ET*-to-daily *ET* ratio (*ET*_*h*_*/ET*_*d*_) variation using *EC* measurements.

**Figure A22.**
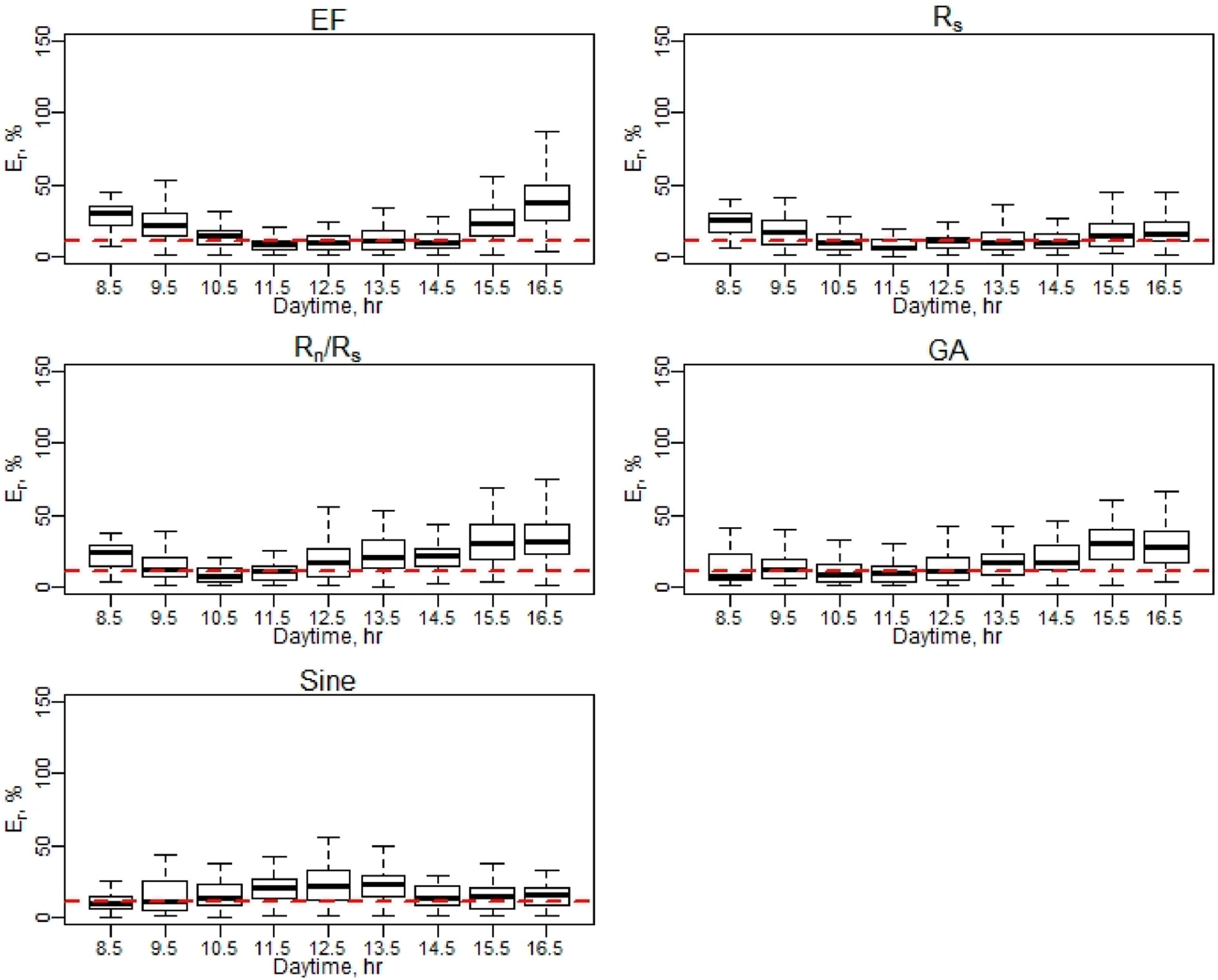
*E*_*r*_ of daily *EC ET* (April–May).

**Figure A23.**
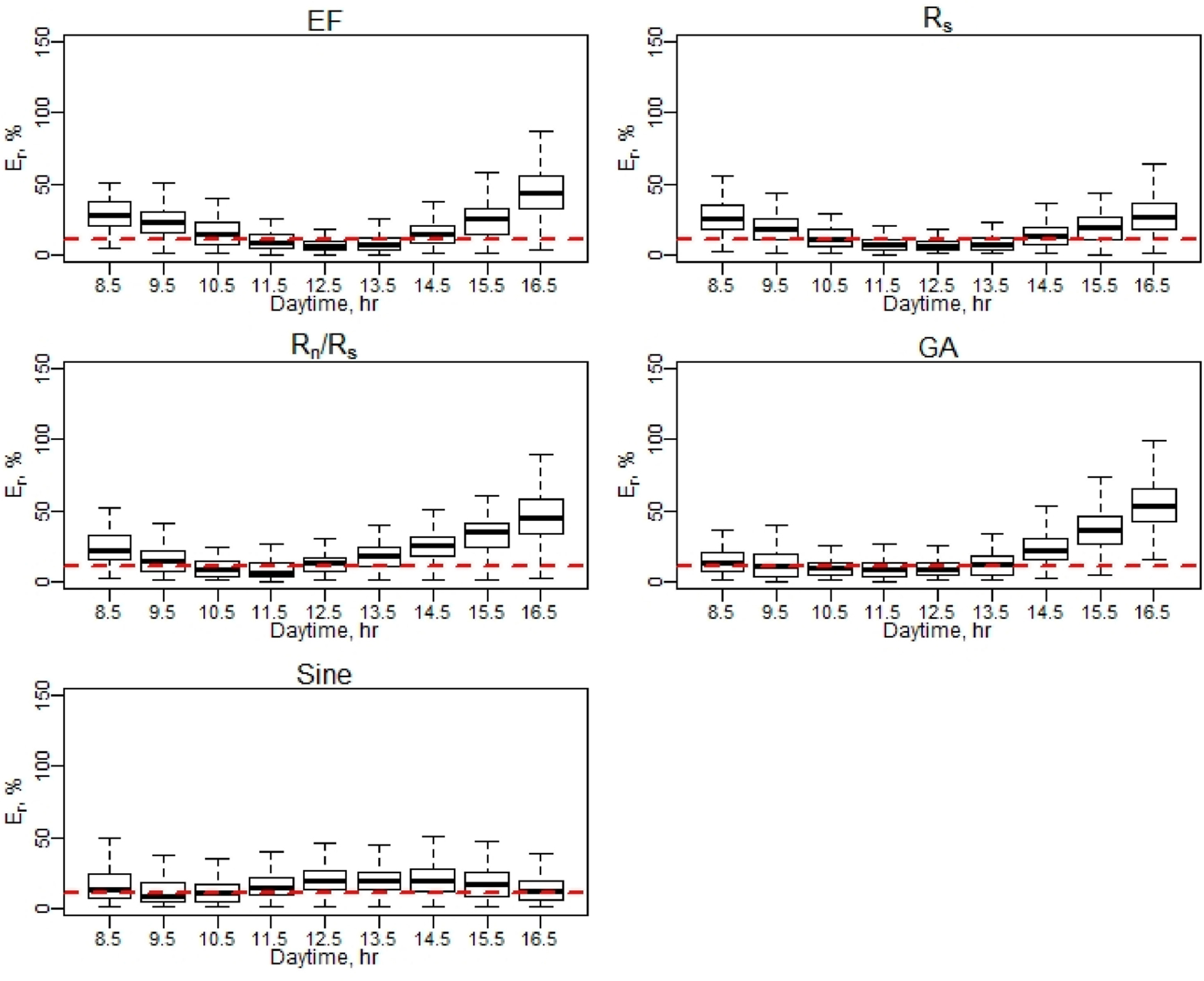
*E*_*r*_ of daily *EC ET* (June–August).

**Figure A24.**
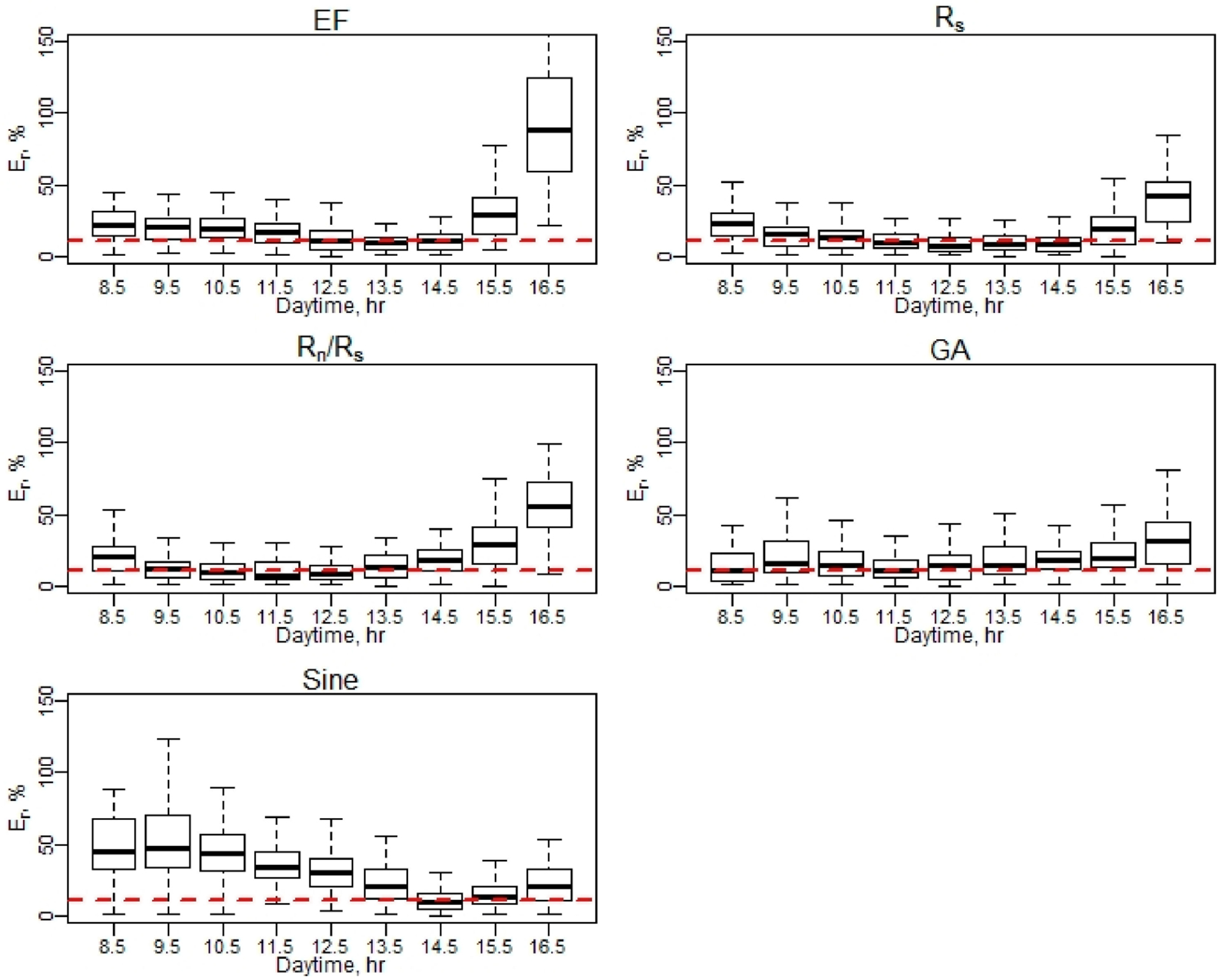
*E*_*r*_ of daily *EC ET* (September–October). Note: Red dash line represents a 10% relative error (*E*_*r*_).

**Figure A25.**
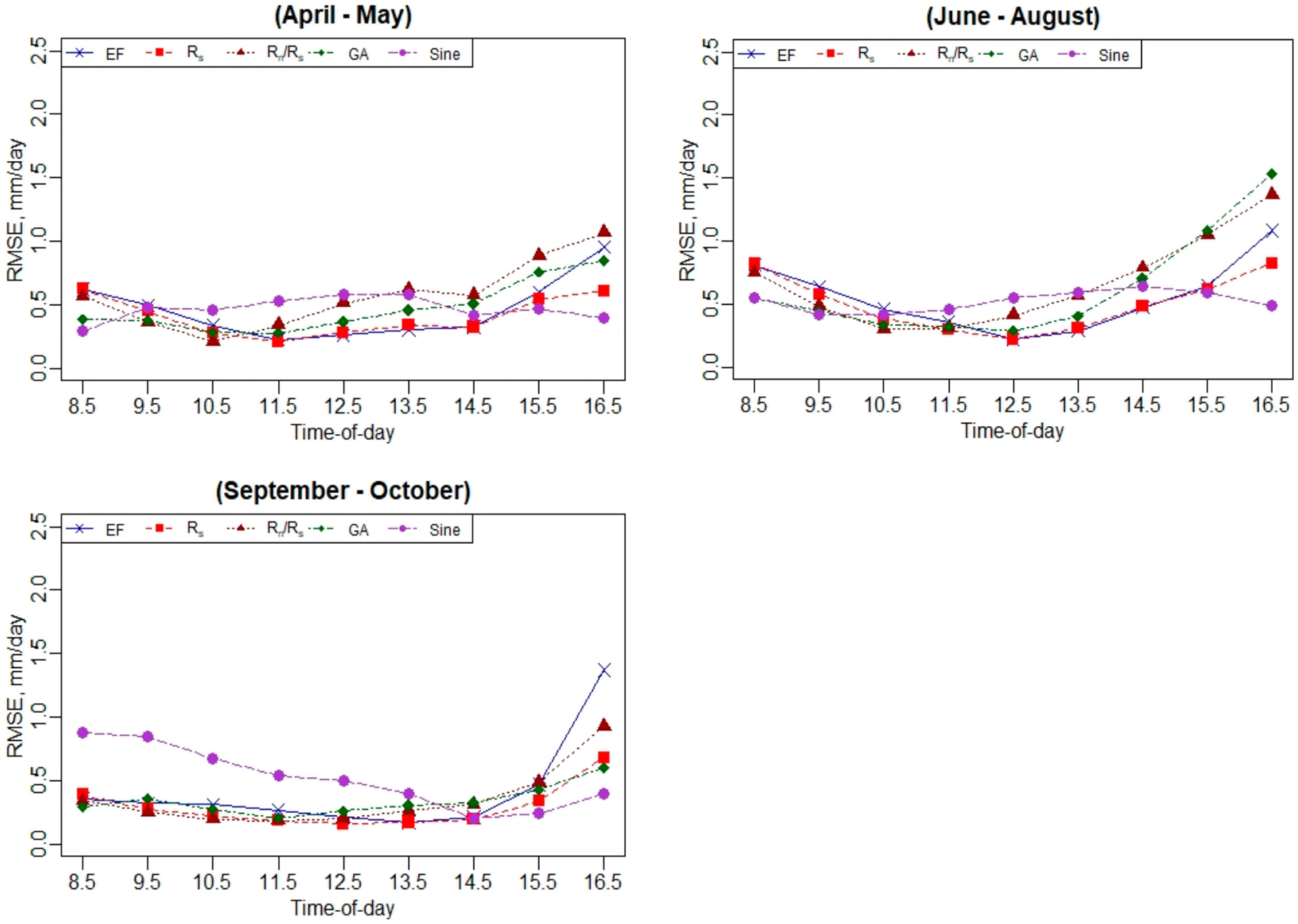
Daily *RMSE* performance using hourly *EC ET* values.
